# JIP4 is recruited by the phosphoinositide-binding protein Phafin2 to promote recycling tubules on macropinosomes

**DOI:** 10.1242/jcs.258495

**Published:** 2021-07-20

**Authors:** Kia Wee Tan, Viola Nähse, Coen Campsteijn, Andreas Brech, Kay Oliver Schink, Harald Stenmark

**Affiliations:** 1 Centre for Cancer Cell Reprogramming, Faculty of Medicine, University of Oslo, Montebello N-0379 Oslo, Norway; 2 Department of Molecular Cell Biology, Institute for Cancer Research, Oslo University Hospital, Montebello 0379 Oslo, Norway; 3 Department of Molecular Medicine, Institute of Basic Medical Sciences, Faculty of Medicine, University of Oslo, 0372 Oslo, Norway

**Keywords:** Endosomes, Macropinocytosis, Membrane recycling, Trafficking

## Abstract

Macropinocytosis allows cells to take up extracellular material in a non-selective manner into large vesicles called macropinosomes. After internalization, macropinosomes acquire phosphatidylinositol 3-phosphate (PtdIns3P) on their limiting membrane as they mature into endosomal-like vesicles. The molecular mechanisms that underlie recycling of membranes and transmembrane proteins from these macropinosomes still need to be defined. Here, we report that JIP4 (officially known as SPAG9), a protein previously described to bind to microtubule motors, is recruited to tubulating subdomains on macropinosomes by the PtdIns3P-binding protein Phafin2 (officially known as PLEKHF2). These JIP4-positive tubulating subdomains on macropinosomes contain F-actin, the retromer recycling complex and the retromer cargo VAMP3. Disruption of the JIP4–Phafin2 interaction, deletion of Phafin2 or inhibition of PtdIns3P production by VPS34 impairs JIP4 recruitment to macropinosomes. Whereas knockout of JIP4 suppresses tubulation, its overexpression enhances tubulation from macropinosomes. JIP4-knockout cells display increased retention of macropinocytic cargo in both early and late macropinosomes. Collectively, these data identify JIP4 and Phafin2 as components of a tubular recycling pathway that operates from macropinosomes.

This article has an associated First Person interview with the first author of the paper.

## Abstract (precis)

**Summary:** The motor adaptor JIP4 is recruited to macropinosomes by the PtdIns3P-binding protein Phafin2 to promote recycling to the plasma membrane via tubular extensions of macropinosomes.

## INTRODUCTION

Macropinocytosis is a process that enables cells to take up large amounts of extracellular fluid (Swanson and King, 2019). This fluid is internalized into large vesicles that are called macropinosomes. During this process, large regions of plasma membrane as well as the proteins within are internalized. Newly formed macropinosomes acquire markers of early endosomes, such as RAB5 and phosphatidylinositol 3-phosphate (PtdIns3P) on their limiting membranes. To preserve the composition of the plasma membrane, it is important that membranes and membrane proteins are recycled from this compartment, and transported back to the cell surface.

After internalization, macropinosomes frequently tubulate and bud off small vesicles (King and Kay, 2019). Tubulation from vesicle membranes often requires the action of membrane-bending proteins, such as sorting nexins (van Weering and Cullen, 2014). In addition, tubulation and the formation of vesicles typically require motor proteins, which exert pulling forces on the nascent membrane tubule. Often, multiple motor proteins are involved in a ‘tug of war’, and this is proposed to generate forces that drive scission of the membrane (Castro-Castro et al., 2016). This motor-driven tubule pulling and scission requires adaptor proteins, which link motor proteins to the tubule membrane.

JIP4 (also known as SPAG9) is a coiled-coil protein that can bind to both dynein and kinesin motor protein complexes (Montagnac et al., 2009; Vilela et al., 2019), and has been functionally linked to organelle positioning (Boecker et al., 2021; Gowrishankar et al., 2021; Willett et al., 2017), endolysosomal membrane reshaping (Marchesin et al., 2015) and recycling (Montagnac et al., 2011). It can also bind to the small GTPase ARF6 (Isabet et al., 2009). JIP4 has been reported to regulate transport of recycling endosomes during cytokinesis, which requires it to interact with ARF6 (Montagnac et al., 2009). ARF6 and JIP4 have also been shown to regulate fast recycling of the transferrin receptor (Montagnac et al., 2011), and are involved in endosomal recycling of the matrix metalloproteinase MMP14 (also known as and hereafter referred to as MT1-MMP) (Marchesin et al., 2015). JIP4 is recruited to the lysosome upon lysosomal damage by phosphorylated RAB10, where it triggers microtubule-dependent tubulation, a process called lysosomal tubulation driven by LRRK2 (LYTL) (Bonet-Ponce et al., 2020). The transmembrane protein PIP4P1 (also known as TMEM55B) recruits JIP4 to lysosomes to mediate long-distance lysosome transport (Willett et al., 2017).

Here, we show that JIP4 is recruited to retromer-containing tubules of tubulating macropinosomes by the lipid-binding protein Phafin2 (also known as PLEKHF2) in a PtdIns3P-dependent fashion. The PH domain of Phafin2 binds to a so-far undescribed and poorly conserved region on JIP4, and ablation of JIP4 results in a retention of fluid-phase cargo in early and late endosomal compartments. These results suggest that Phafin2 recruits JIP4 to newly internalized macropinosomes where it promotes membrane tubulation and recycling.

## RESULTS

### JIP4 interacts with Phafin2 *in vitro*

**Fig. 1.**
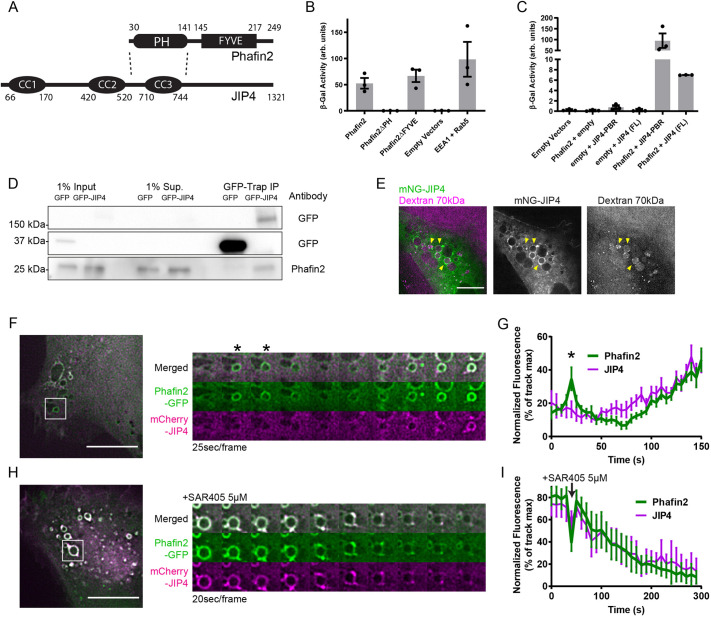
**JIP4 interacts and colocalizes with Phafin2.** (A) Domain structure of JIP4 and Phafin2, dotted lines indicate interacting regions. CC1, 2 and 3 indicate predicted coiled coil regions. (B) β-galactosidase activity derived from yeast two-hybrid assay of cells expressing the specified constructs, with JIP4 Phafin2-binding-region as prey. Mean±s.e.m. (C) β-galactosidase activity derived from yeast two-hybrid assay of cells expressing the specified constructs, with full length Phafin2 as bait. FL, full length; PBR, Phafin2-binding-region. Mean±s.e.m. (D) Immunoprecipitation of GFP-JIP4 with GFP-Trap, western blotting against GFP and endogenous Phafin2 in RPE-1 lysate. Uncropped blots are shown in Fig. S1C,D. Shown are representative blots of three experiments. (E) RPE-1 cell expressing mNeonGreen-JIP4 and labeled with 70 kDa dextran-TexasRed. Yellow arrowheads highlight dextran-filled vesicles positive for JIP4. (F) Live imaged RPE-1 cell expressing Phafin2-GFP and mCherry-JIP4. Montage gallery of the boxed region is shown on the right. Asterisks highlight Phafin2 localization at a nascent macropinosome. (G) Mean fluorescence measurements along the limiting membrane of macropinosomes, treated as in G. Each measurement was normalized against the mean of the individual time series, aligned at timepoint 15 s to the peak of Phafin2 fluorescence on nascent macropinosomes (*), ±95% C.I. (*n*=13 macropinosomes). (H) Live imaged RPE-1 cell expressing Phafin2-GFP and mCherry-JIP4, treated with the VPS34 inhibitor SAR405 to remove PtdIns3P from macropinosomes. Montage gallery of the boxed region is shown on the right. (I) Mean fluorescence measurements along the limiting membrane of macropinosomes treated as in I. Each measurement was normalized to the mean of the individual time series ±95% C.I. (*n*=17 macropinosomes). Scale bars: 10 µm.

We have recently identified the phosphoinositide-binding protein Phafin2 to be a regulator of macropinosome formation (Schink et al., 2017 preprint). By using a two-hybrid screen for Phafin2 interactors, we identified JIP4 as a potential interactor of Phafin2 (Table S1). This was interesting, as JIP4 and its homolog JIP3 had previously been reported to be important for macropinocytosis (Williamson and Donaldson, 2019). We first confirmed the interaction of JIP4 with Phafin2 by using yeast two-hybrid interaction assays with truncation mutants of Phafin2 against the identified interaction region within JIP4 at amino acids (aa) 566–767 (Fig. 1A). For brevity, we have labeled our figures to denote this JIP4 region as the Phafin2-binding region (PBR). Phafin2 contains a PH and a FYVE domain, both of which are involved in lipid binding (Fig. 1A) (Matsuda-Lennikov et al., 2014; Schink et al., 2017 preprint; Tang et al., 2017, 2020). JIP4 interacts with Phafin2 only through the Phafin2 PH domain, as deletion of the PH domain – but not the FYVE domain – abolished expression of the reporter gene in a yeast two-hybrid assay (Fig. 1B). To extend these results to mammalian cells, we performed proximity biotinylation labeling by using cell lines stably expressing APEX2-fusions of full length or deletion mutants of Phafin2, with cell lines expressing cytosolic or membrane-anchored APEX2 serving as negative controls. Semi-quantitative mass spectrometry analysis showed that deletion of the Phafin2 PH domain greatly impaired biotinylation of JIP4, whereas deletion of the FYVE domain – that is required for localization of Phafin2 to early macropinosomes (Schink et al., 2017 preprint) – did not (Fig. S1A, Table S2). Together, these experiments indicate that the PH domain of Phafin2 is involved in interaction with JIP4, whereas the FYVE domain is not.

To verify that full-length JIP4 is also capable of interacting with Phafin2, we used yeast two-hybrid assays and immunoprecipitation. Full-length JIP4, like the isolated interaction region previously identified, induced expression of the reporter gene in the yeast two-hybrid assay (Fig. 1C). To assess the interaction between Phafin2 and JIP4 in mammalian cells, we performed tandem affinity purification using lysates from RPE-1 cells stably expressing localization and affinity purification (LAP)-tagged Phafin2. Semi-quantitative mass spectrometry analysis identified JIP4 as a strong interactor in these pulldown assays, with a 28-fold enrichment for JIP4 compared with control cells expressing solely the LAP tag (Fig. S1B, Table S3). By contrast, we precipitated GFP-tagged JIP4 (GFP-JIP4) from the cell lysate of human retinal pigment epithelial (RPE-1) cells stably expressing GFP-JIP4 by using GFP-TRAP magnetic beads. Immunoblotting with antibody against Phafin2 showed that endogenous Phafin2 co-precipitated with GFP-JIP4 but not with GFP alone (Fig. 1D; Fig. S1C,D). We therefore conclude that JIP4 interacts with Phafin2 both *in vitro* and within cells.

### JIP4 dynamically colocalizes with Phafin2 to macropinosomes

To assess the subcellular distribution of JIP4, we tagged JIP4 with mNeonGreen (mNG-JIP4) and expressed it in RPE-1 cells. mNG-JIP4 localized to the limiting membrane of large vesicles, which were positive for 70 kDa dextran added to the extracellular medium (Fig. 1E). These JIP4-positive structures are, therefore, likely to be macropinosomes that contain fluid-phase cargo endocytosed from the extracellular medium.

In our previous study, we have found that Phafin2 is recruited to macropinosomes (Schink et al., 2017 preprint), and the *in vitro* data above suggested that JIP4 and Phafin2 interact on the macropinosome membrane. We therefore performed live-cell microscopy to assess the dynamic localization of JIP4 and Phafin2. As previously reported, Phafin2 showed a distinctive biphasic localization to macropinosomes, i.e. one to nascent macropinosomes, directly after scission from the membrane, and one to macropinosomes that have matured into endosome-like vesicles (Schink et al., 2017 preprint). In our current study, we refer to these structures as early macropinosomes because they acquire markers of early endosomes. JIP4 accumulated together with Phafin2 at early macropinosomes but did not colocalize with Phafin2 to nascent macropinosomes (Fig. 1F,G; Movie 1).

Phafin2 requires PtdIns3P generated by the PtdIns 3-kinase VPS34 to localize to early macropinosomes and endosomes (Schink et al., 2017 preprint). To test whether JIP4 localization to macropinosomes is also dependent on PtdIns3P, we treated cells expressing tagged JIP4 and Phafin2 with the selective VPS34 inhibitor SAR405 (Ronan et al., 2014) and assessed the localization of JIP4 using live-cell microscopy. Inhibition of the production of PtdIns3P by VPS34 led to a concurrent and rapid displacement of both Phafin2 and JIP4 from the membrane (Fig. 1H,I). The direct interaction of JIP4 with Phafin2 and their simultaneous displacement from the membrane suggested that Phafin2 functions as a recruiter of JIP4 to the early macropinosome.

**Fig. 2.**
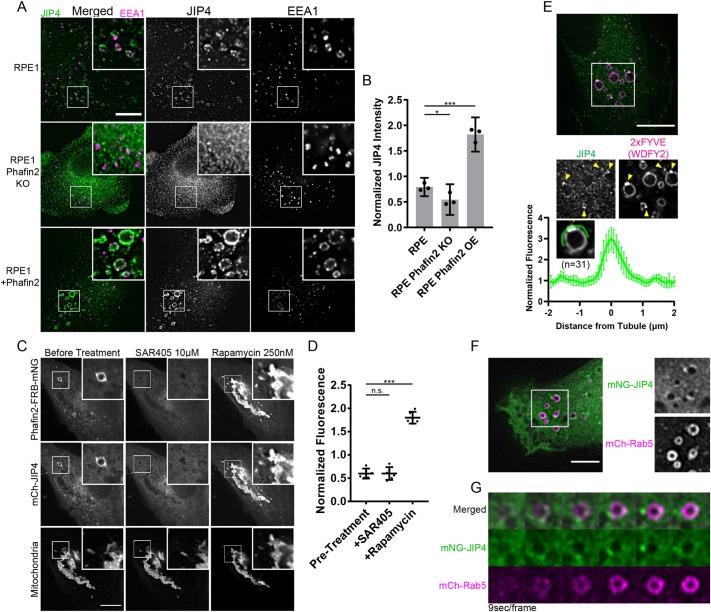
**Membrane recruitment of JIP4 by Phafin2.** (A) Representative images of RPE-1 cells of the specified genotypes, fixed and immunostained against JIP4 and EEA1. Brightness settings are equal across all images and magnifications. Boxed areas are shown magnified at top right of each image. (B) Mean intensities of JIP4 immunostaining inside EEA1 positive vesicles, each experiment was normalized against the mean of all datapoints in that experiment. Shown is the mean of three experiments, ±95% C.I. (3530-6121 vesicles per condition per experiment). (C) RPE-1 cell expressing Phafin2-FRB-mNeonGreen, mCherry-JIP4, and mitochondrion-anchored mTagBFP2 2×FKBP (mitochondria). Shown are images of the same cell before addition of 10 µM of the VPS34 inhibitor SAR405 (left), after SAR405 has removed macropinosomal PtdIns3P (middle) and after 250 nM rapamycin has recruited Phafin2 to the mitochondrial membrane (right). Boxed areas are shown magnified at top right of each image. (D) JIP4 fluorescence at mitochondria, fluorescence was acquired of the same cells under the three sequential conditions, segmented and measured using the mitochondrial marker as shown in C. Error bars are 95% C.I. (*n*=6 cells). (E) Representative image of an RPE-1 cell expressing 2xFYVE^(WDFY2)^ as a marker for PtdIns3P, fixed and immunostained against JIP4. The boxed area of the top image is shown magnified in grayscale below. Yellow arrowheads mark membrane deformations. Plotted below is the normalized fluorescence intensity across a region measured as in the example ROI (green), mean±95% C.I. (*n*=31 macropinosomes). (F) Representative image of a live imaged RPE-1 cell expressing mNeonGreen-JIP4 and mCherry-Rab5. The boxed area is shown magnified in grayscale on the right. (G) Montage gallery of a macropinosome as it matures into a Rab5-positive early macropinosome and acquires JIP4. Scale bars: 10 µm

If being a putative recruiter, modulation of Phafin2 protein levels by overexpression or ablation would be expected to affect JIP4 localization. We assessed endogenous JIP4 localization to early endosomes in wild-type, Phafin2-KO (Schink et al., 2017 preprint) or Phafin2-overexpressing RPE-1 cells by immunostaining for JIP4 and the early-endosomal antigen EEA1, and by quantifying JIP4 intensity in EEA1-labeled endosomes. We found reduced localization of endogenous JIP4 to early endosomes in Phafin2-KO cells. By contrast, overexpression of Phafin2 led to strong recruitment of JIP4 to EEA1-positive endosomes (Fig. 2A,B), which is consistent with the notion that Phafin2 recruits JIP4.

To further support that JIP4 is recruited by Phafin2, we used a chemical dimerization system to redirect Phafin2 to mitochondria and monitored the localization of JIP4. To this end, we expressed FRB- and fluorophore-tagged Phafin2, a mitochondrion-anchored 2xFKBP domain (Tom70-mTagBFP2-2xFKBP), and fluorophore-tagged JIP4 in RPE-1 cells. FK506-binding protein (FKBP) and FKBP-rapamycin-binding (FRB) domains heterodimerize in the presence of rapamycin (Putyrski and Schultz, 2012), allowing redirection of FRB-tagged Phafin2 to the mitochondria by adding rapamycin to the extracellular solution. Cells expressing all three components were first treated with SAR405, to release Phafin2 and JIP4 from macropinosomes (Fig. 2C,D). Addition of rapamycin caused FRB-tagged Phafin2 to be recruited to mitochondria (Fig. 2C,D). Importantly, JIP4 was recruited together with Phafin2 to the mitochondria, indicating that Phafin2 does not require additional macropinosome co-factors to bind and recruit JIP4.

Early macropinosomes are rich in PtdIns3P. We expressed a tandem FYVE-domain phosphoinositide probe derived from WDFY2 [2xFYVE^(WDFY2)^] to mark PtdIns3P-positive membranes in RPE-1 cells and immunostained against JIP4. This FYVE probe is able to bind to PtdIns3P-containing membranes of different curvatures and, therefore, allows visualization of membrane subdomains (Sneeggen et al., 2019). Endogenous JIP4 was found localized to PtdIns3P-positive vesicular compartments (Fig. S2). This JIP4 localization was more prominent at areas where the vesicle membrane appeared deformed, and quantification of JIP4 intensities showed clear enrichment at these regions (Fig. 2E). Live-cell microscopy of mNG-JIP4 together with the early endosome protein RAB5 showed that JIP4 is acquired on the macropinosome as it matures into a RAB5-positive structure (Fig. 2F,G; Movie 2), and that it associated in subdomains on the early macropinosome membrane.

### JIP4-Phafin2 interaction is not conserved in homologs

**Fig. 3.**
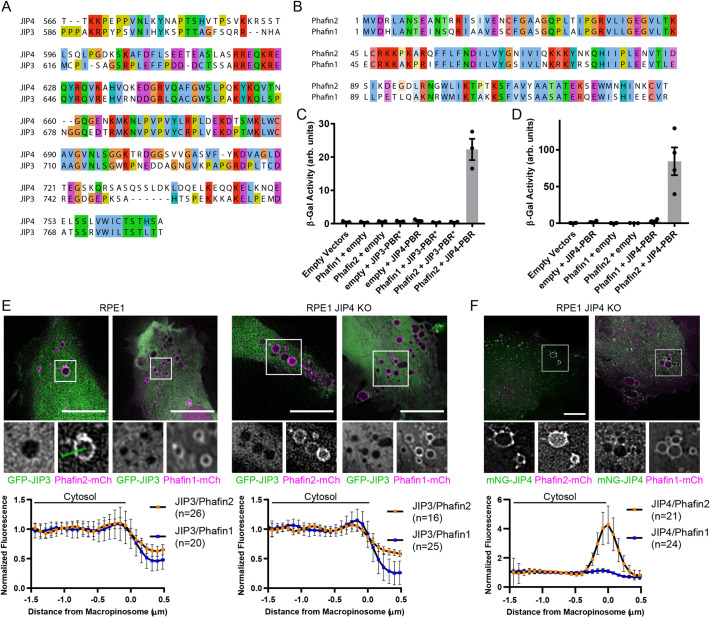
**JIP4-Phafin2 interaction is not conserved in homologs.** (A) Alignment of the JIP4 Phafin2-binding-region (PBR) with the homologous JIP3 region (PBR*). Colors are according to the ClustalX scheme for physicochemical properties. (B) Alignment of the Phafin2 and Phafin1 PH domains. Colors are according to the ClustalX scheme. (C) β-galactosidase activity derived from yeast two-hybrid assay of cells expressing the specified constructs. PBR, Phafin2-binding region, PBR*, JIP3 region homologous to PBR. Mean±s.e.m. (D) β-galactosidase activity derived from yeast two-hybrid assay of cells expressing the specified constructs. PBR: Phafin2-binding-region. Mean±s.e.m. (E) Representative images of cells of the indicated genotypes expressing GFP-JIP3 and a Phafin isoform. JIP3 is not recruited to macropinosomes. The boxed area is shown magnified below. Plotted below is the normalized fluorescence intensity measured across as in the example line ROI, mean±95% C.I. (F) Representative JIP4 KO cells expressing mNeonGreen-JIP4 and a Phafin isoform. Phafin1 does not recruit JIP4. The boxed area is shown magnified below. Plotted below is the normalized fluorescence intensity measured across as in the example line ROI in E (boxed), mean±95% C.I. Scale bars: 10 µm.

Both Phafin2 and JIP4 have homologs in the human genome, Phafin1 and JIP3, which share a large degree of sequence homology (Fig. 3A,B; Fig. S3A,B). It is often implied that JIP3 and JIP4 have similar functions (Marchesin et al., 2015; Sato et al., 2015; Vilela et al., 2019; Williamson and Donaldson, 2019). We therefore asked whether these homologs can functionally replace each other. First, we tested whether Phafin1 or Phafin2 can bind to JIP3 by using direct two-hybrid interaction assays. To this end, we isolated the region corresponding to the identified JIP4-Phafin2 interaction domain from JIP3 based on the JIP3/JIP4 sequence homology. We did not observe any interaction, neither between Phafin1 and JIP3 nor between Phafin2 and JIP3 (Fig. 3C). We also tested whether Phafin1 can bind to JIP4 in two-hybrid interaction assays. Despite the high sequence homology between the PH domains of Phafin1 and Phafin2 (Fig. 3B), we did not observe any interaction between Phafin1 and JIP4 (Fig. 3D). This suggests that the interaction between Phafin2 and JIP4 is isoform specific.

To confirm the data obtained through two-hybrid interaction assays and to verify that the full-length proteins do not contain interaction sites outside the regions analyzed in two-hybrid assays, we coexpressed different combinations of Phafin1/2 and JIP3/4 in RPE-1 cells. JIP3 and JIP4 dimerize through coiled-coil regions (Isabet et al., 2009; Llinas et al., 2016; Vilela et al., 2019), form heterodimers in cells and could be recruited together. To account for this, we expressed GFP-tagged JIP3 together with either Phafin2 or Phafin1 in both wild-type cells and cells that lack endogenous JIP4 (Fig. S3C–E), and assayed the localization of JIP3 (Fig. 3E). Whereas Phafin1 – similarly to Phafin2 – localized to macropinosomes, we did not observe any localization of JIP3 to these vesicles (Fig. 3E). By contrast, when mNeonGreen-JIP4 was expressed together with either Phafin2 or Phafin1 in cells depleted for endogenous JIP4, it was readily recruited to macropinosomes by Phafin2 but not Phafin1 (Fig. 3F). Taken together, these data show that only Phafin2 interacts with JIP4, whereas its homolog Phafin1 does not bind to JIP4. Moreover, the JIP4 homolog JIP3 is unable to bind to either Phafin2 or Phafin1.

### Formation of macropinosomes does not require JIP4

**Fig. 4.**
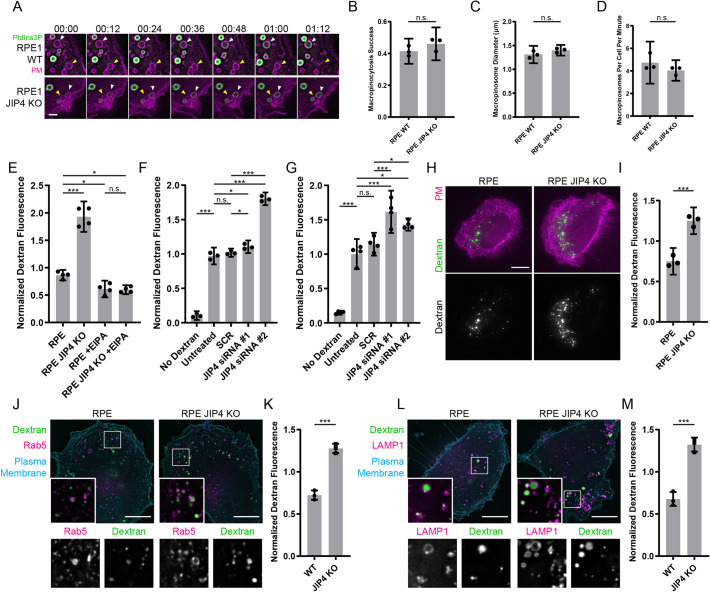
**JIP4 ablation causes accumulation of fluid-phase cargo.** (A) Time-lapse images of RPE-1 cells expressing mNeonGreen-2xFYVE as a PtdIns3P marker and Myrpalm-mCherry as a plasma membrane marker. Yellow arrowheads indicate a macropinosome that fails to mature to an early macropinosome and fuses again with the plasma membrane. White arrowheads indicate a macropinosome that matures to an early macropinosome and acquires 2xFYVE. Scale bar: 1 µm. (B) Fraction of macropinosomes per cell that successfully mature into an early macropinosome. The mean of three experiments is shown. Error bars are 95% C.I. (10–15 cells per genotype per experiment). (C) Diameter of newly formed 2xFYVE-positive macropinosomes. The mean of three experiments is shown. Error bars are 95% C.I. (19–28 cells per genotype per experiment). (D) Frequency of newly formed 2xFYVE-positive macropinosomes of the indicated genotypes. The mean of three experiments is shown. Error bars are 95% C.I. (19–28 cells per genotype per experiment). (E) Median fluorescence of 2×10^4^ RPE-1 cells after 30 min uptake of fluorescent 10 kDa dextran, measured by flow cytometry. Shown is the mean±95% C.I. of four experiments. (F) Median fluorescence of 10^4^ RPE-1 cells after 30 min uptake of 70 kDa dextran-TexasRed, measured by flow cytometry. Shown is the mean±95% C.I. of three experiments. (G) Median fluorescence of 10,000 HT1080 cells after 30 min uptake of 70 kDa dextran-TexasRed, measured by flow cytometry. Shown is the mean±95% C.I. of four experiments. (H) Representative images of RPE-1 cells of the indicated genotype after 30 min uptake of fluorescent 10 kDa dextran. A plasma membrane marker is shown in magenta. Scale bar: 5 µm. (I) Total dextran fluorescence per cell of the indicated genotypes after a 30 min uptake of dextran. Shown is the mean±95% C.I. of three experiments (15–20 cells per genotype per experiment). (J) RPE-1 cell of the indicated genotypes expressing mCherry-Rab5 after a 30 min uptake of fluorescent 10 kDa dextran. Boxed areas are shown magnified at bottom left of and below each image. Scale bars: 5 µm. (K) Dextran fluorescence in Rab5-positive compartments per cell of the indicated genotype after 30 min uptake of 10 kDa dextran. Shown is the mean±95% C.I. of three experiments (15-20 cells per genotype per experiment). (L) Representative images of RPE-1 cells of the indicated genotypes expressing mCherry-LAMP1 after a 30 min uptake of 10 kDa dextran. Boxed areas are shown magnified at bottom left of and below each image. Scale bars: 5 µm. (M) Dextran fluorescence in LAMP1-positive compartments per cell of the indicated genotype after 30 min uptake of dextran. Shown is the mean±95% C.I. of three experiments (15–20 cells per genotype per experiment).

We have previously shown that Phafin2 is involved in the formation of macropinosomes (Schink et al., 2017 preprint), and JIP3 and JIP4 have been proposed to influence macropinocytosis (Williamson and Donaldson, 2019). Therefore, we tested whether JIP4 is required to form macropinosomes from membrane ruffles. By tracking individual macropinosomes and measuring whether they successfully mature into early macropinosomes, we found that loss of JIP4 does not affect early steps of macropinocytosis (Fig. 4A,B). Early macropinosomes formed in JIP4 KO cells did not differ in size or frequency to wild-type cells (Fig. 4C,D). During macropinocytosis, JIP4 therefore appears to act after the internalization process is complete and the vesicle has firmly gained endosomal membrane identity – as evidenced by the accumulation of PtdIns3P on the limiting membrane.

### JIP4 knockout causes intracellular accumulation of fluid-phase cargo

A distinguishing feature of macropinocytosis as an endocytic route is that it is efficient at internalizing extracellular fluid. To investigate the functional role of JIP4 at macropinosomes, we used flow cytometry to compare the internalization of extracellular fluid of wild-type RPE-1 cells and RPE-1 JIP4-knockout (KO) cells by using 10 kDa dextran as a fluid-phase marker. JIP4-KO cells showed significantly elevated intracellular dextran levels in comparison to wild-type cells after a 30 min uptake period (Fig. 4E). Blockage of macropinocytic uptake by using the macropinocytosis inhibitor 5-(N-ethyl-N-isopropyl) amiloride (EIPA) (Meier et al., 2002) abolished this phenotype. Similarly, knockdown (KD) of JIP4 in RPE-1 and HT1080 cells using two different siRNAs (Fig. S4A,B) and 70 kDa dextran resulted in significantly elevated intracellular dextran levels in comparison to wild-type cells (Fig. 4F,G). We also performed a similar assay to measure dextran fluorescence in wild-type and JIP4 KO RPE-1 cells by using microscopy and 10 kDa dextran, which showed that JIP4 KO cells contained more dextran than wild-type cells after 30 min of dextran labeling (Fig. 4H,I).

As the ablation of JIP4 neither impaired nor improved macropinocytosis, the increased intracellular dextran levels suggested that processing of internalized intracellular material had been altered. We generated stable wild-type and JIP4 KO cell lines expressing RAB5 to label early macropinosomes and LAMP1 to label late macropinosomes and measured dextran intensity within these compartments.

In order to label early and late macropinosomes, we generated wild-type and JIP4 KO cell lines that stably expressed RAB5 and LAMP1, respectively, and measured dextran intensity in both compartments. In line with our previous findings, we observed increased dextran fluorescence in both RAB5- (Fig. 4J,K) and LAMP1-positive (Fig. 4L,M) – i.e. early and late – macropinosomes, suggesting that more dextran is retained in either macropinosome when JIP4 is absent.

### JIP4 is targeted by Phafin2 to macropinosome tubules

**Fig. 5.**
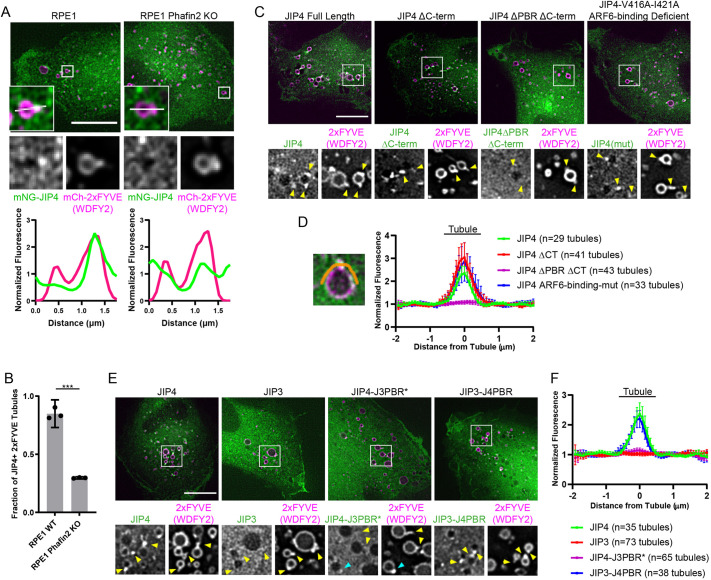
**JIP4 is recruited to macropinosome tubules by Phafin2.** (A) Representative images and magnifications of RPE-1 cells of the specified genotype expressing mNeonGreen-JIP4 and mCherry-2xFYVE^(WDFY2)^. Line plots are taken along the indicated line from left to right. Boxed areas are shown magnified at bottom left of and below each image. Scale bars: 10 µm. (B) Fraction of 2xFYVE^(WDFY2)^ tubules per cell positive for mNG-JIP4. The positive threshold was set at 1.5× cytoplasmic fluorescence. Shown is the mean±95% C.I. of three experiments (42-105 tubulation events per genotype per experiment). (C) Representative images of RPE-1 JIP4 KO cells expressing 2xFYVE^(WDFY2)^ as a PtdIns3P marker and the indicated JIP4 construct. Yellow arrowheads indicate tubules. Boxed areas are shown magnified below each image. Scale bar: 10 µm. (D) Example line ROI is shown in orange. Plotted is the normalized fluorescence intensity measured across as in the example ROI, mean±95% C.I. (E) Representative images of RPE-1 JIP4 KO cells expressing 2xFYVE^(WDFY2)^ as a PtdIns3P marker and the indicated JIP construct. Yellow arrowheads indicate tubules. Cyan arrowhead indicates an example of a tubule positive for the JIP4-J3PBR* chimera (highlighted in Results and Discussion), this data point was included in the quantification shown in F. Boxed areas are shown magnified at bottom left of and below each image. Scale bar: 10 µm. (F) Plotted is the normalized fluorescence intensity measured across as in the example line ROI (indicated in D), mean±95% C.I.

As described above, JIP4 localizes to subdomains on RAB5-positive early macropinosomes. Live-cell imaging of mNG-JIP4 together with 2xFYVE^(WDFY2)^-labeling of PtdIns3P-positive membranes showed that JIP4 preferentially localized to highly dynamic membrane deformations from which membrane tubules emerged (Movie 3). In wild-type RPE-1 cells, JIP4 localized to mCherry-2xFYVE^(WDFY2)^ labeled tubules, but this localization was largely lost in cells depleted of the JIP4 recruiter Phafin2 (Fig. 5A,B). To assess the importance of JIP4 binding to Phafin2 for its tubule localization without disrupting Phafin2 (and other associated functions), we constructed various mutants of JIP4 (Fig. S5A) and compared their localizations in live-cell imaging with 2xFYVE^(WDFY2)^ to label PtdIns3P-positive membranes. These experiments were carried out in RPE-1 JIP4 KO cells to prevent dimerization with endogenous JIP4. Full-length JIP4 localized to tubular membranes as described previously, as did a JIP4 construct truncated after the Phafin2-binding region (mNG-JIP4 ΔCT) (Fig. 5C,D). Extending the truncation to remove the PBR (mNG-JIP4 ΔPBR ΔCT) abolished the localization to tubules (Fig. 5C,D). As ARF6 had previously been reported to be involved in JIP4 function, we also tested a JIP4 mutant containing the mutations V416A and I421A. It had been shown previously that these mutations strongly reduce binding to ARF6 (27-fold and 18-fold, respectively) (Isabet et al., 2009). Interestingly, we found the mNG-JIP4-V416A-I421A construct to be robustly associated with membrane tubules (Fig. 5C,D). This indicates that the Phafin2-binding region on JIP4 is crucial to target JIP4 onto macropinosome tubules in a manner that is independent of ARF6 binding, which occurs on a separate part of JIP4 (Montagnac et al., 2009).

To further investigate the difference between JIP3 and JIP4, we constructed a chimera of each homolog. The PBR of JIP4 was moved into JIP3 yielding JIP3-J4PBR, and the PBR-like region (PBR*) of JIP3 into JIP4 yielding JIP4-J3PBR*. The swapped regions were extended by one aa to retain the local sequence environment, such that each started and ended on the same aa than their respective isoform (Fig. S5B). We then compared the localizations of wild-type JIP4, wild-type JIP3, IP4-J3PBR* and JIP3-J4PBR by using live-cell microscopy. As previously, this experiment was carried out in JIP4 KO RPE-1 cells, and with 2xFYVE^(WDFY2)^ to label PtdIns3P-positive membranes. JIP4 and JIP3-J4PBR robustly localized to macropinosome tubules (Fig. 5E,F). By contrast, JIP3 did not localize at all to macropinosome tubules, and JIP4-PBR* only showed rare and extremely weak localization to tubules (Fig. 5E,F). Taken together, these experiments indicate that JIP4, when binding Phafin2 through the PBR region, confers its ability to target macropinosome tubules and that the homologous region on JIP3 is unable to do so.

### JIP4-positive recycling tubules are extruded from macropinosomes

**Fig. 6.**
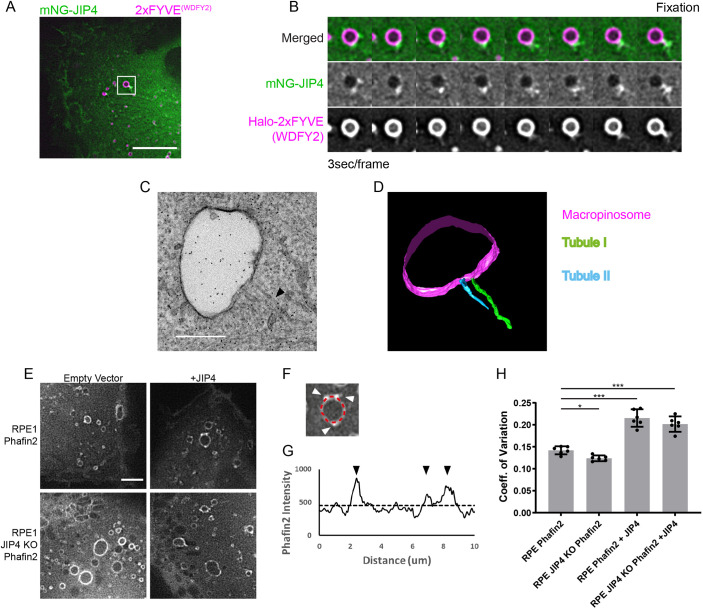
**JIP4 tubules are extruded and continuous with macropinosomes.** (A) Image of RPE-1 cell expressing mNeonGreen-JIP4 and Halo-2xFYVE^(WDFY2)^, imaged live during preparation of the CLEM specimen. The boxed area indicates a tubulating macropinosome. Scale bar: 5 µm. (B) Time-lapse montage gallery of the boxed region in A, showing the tubulating macropinosome until fixation with glutaraldehyde. (C) Electron micrograph of the macropinosome depicted in A and B. Black dots are gold fiduciaries for electron tomography. The longest tubule emanating from the JIP4 concentration is marked by a black arrowhead. Scale bar: 500 nm. (D) Model reconstructed from electron tomographs of the macropinosome depicted in C. The limiting membrane of the macropinosome is shown in magenta, two separate emanating tubules are shown in green and blue. The green tubule corresponds to the tubule indicated in C. (E) Representative images of RPE-1 cells of the indicated genotypes expressing the specified constructs. The Phafin2 channel is shown. Scale bar: 5 µm. (F) Example macropinosome, in the Phafin2 channel, depicting the measurement of Phafin2 fluorescence intensity along the limiting membrane of the macropinosome (red dashed line). White arrowheads indicate Phafin2 accumulation at tubule nucleating spots. Notice that the tubule is beginning to extend from the nucleating spot on the top right. (G) Line profile of Phafin2 fluorescence intensity taken along the limiting membrane in F. Black arrowheads indicate the accumulation of Phafin2 as shown in F. The black dashed line indicates the mean of the line plot. (H) Coefficient of variation of Phafin2 fluorescence intensity along line plots taken around macropinosomes >1 µm in diameter as shown in F, of the indicated genotypes. Shown is the mean±95% C.I. of six experiments (21–72 macropinosomes per condition per experiment).

The resolution of light microscopy is unable to reveal membrane ultrastructure. Therefore, to visualize the macropinosome membrane, we performed correlative light and electron microscopy (CLEM). We first followed mNG-JIP4 localization together with Halo-2xFYVE^(WDFY2)^ by live-cell imaging and then chemically fixed the cells during imaging (Fig. 6A,B; Fig. S6A,B). Fixed cells were processed for electron microscopy and micrographs for electron tomography were collected (Fig. 6C; Fig. S6C). Reconstruction of these tomograms showed that the JIP4 accumulations corresponded to membrane tubules extruding from the limiting membrane of the macropinosome (Fig. 6D; Fig. S6D,E).

As we observed accumulation of JIP4 at emerging membrane tubules, we assessed its involvement in tubulation from macropinosomes. We measured tubulation from Phafin2-positive macropinosomes (Fig. 6E–H) in wild-type cells and Phafin2-expressing JIP4-KO cells. In addition, cells were transfected with either empty vector or a JIP4-expressing plasmid. In order to quantify tubulation, we measured the coefficient of variation of the Phafin2 fluorescence over the limiting membrane of the macropinosome (Fig. 6F,G). A higher variation of the fluorescence corresponds to more tubulation events, as these form bright nucleation spots directly at the limiting membrane (Fig. 6F,G). We found that, in comparison to wild-type cells, JIP4 KO cells showed a small but significant reduction of macropinosome tubulation in response to Phafin2 expression (Fig. 6H). By contrast, expression of both Phafin2 and JIP4 in wild-type or KO cells led to a strong increase in macropinosome tubulation (Fig. 6H), suggesting that Phafin2 and JIP4 act together to drive tubulation.

**Fig. 7.**
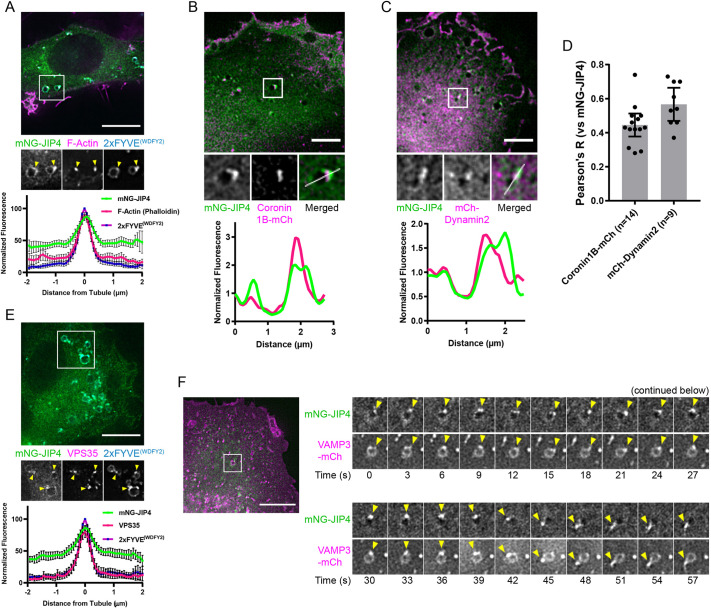
**JIP4 tubules bear markers of membrane recycling zones.** (A) Representative image of RPE-1 cell expressing mNG-JIP4 and 2xFYVE^(WDFY2)^, fixed and stained against F-actin with phalloidin. The boxed area of the top image is shown magnified below; yellow arrowheads indicate tubulating regions. Scale bar: 10 µm. The graph plots normalized fluorescence intensity measured across as in the example ROI in Fig. 4D, mean±95% C.I. (*n*=38 tubules). (B) Representative image of an RPE-1 cell expressing mNeonGreen-JIP4 and coronin1B-mCherry. The boxed area of the top image is shown magnified below. Scale bar: 5 µm. Plotted is the normalized fluorescence intensity measured along the indicated line from left to right. (C) Representative image of RPE-1 cell expressing mNeonGreen-JIP4 and Dynamin2-mCherry. Scale bar: 5 µm. Plotted is the normalized fluorescence intensity measured along the indicated line from left to right. (D) Pearson correlation coefficient (Pearson's R) of the indicated construct against mNG-JIP4. Each datapoint (and *n* number) represents one cell. Mean±95% C.I. (E) Representative image of an RPE-1 cell expressing mNG-JIP4 and 2xFYVE^(WDFY2)^, fixed and stained against VPS35. The boxed area of the top image is shown magnified below. Yellow arrowheads indicate tubulating regions. Scale bar: 10 µm. Plotted is the normalized fluorescence intensity measured as for the ROI example shown in Fig. 4D, mean±95% C.I. (*n*=41 tubules). (F) RPE-1 cell expressing mNeonGreen-JIP4 and VAMP3-mCherry. Scale bar: 5 µm. Time-lapse montage gallery of the boxed region, showing the exit of VAMP3 out of a JIP4-positive tubule (yellow arrowheads). Contrast was not adjusted, the flare between 39 s and 45 s is due to passing debris.

To characterize these macropinosome tubules in more detail, we examined localization of JIP4 together with different recycling markers. We fixed RPE-1 cells that expressed mNG-JIP4 and 2xFYVE^(WDFY2)^, and stained for F-actin using fluorescently labeled phalloidin. JIP4 tubules emerged from actin-rich subdomains on the macropinosome (Fig. 7A). In live-cell microscopy the actin-binding protein coronin1B (Fig. 7B,D) and the large GTPase dynamin-2 (Fig. 7C,D) colocalized with JIP4-positive structures. JIP4-positive tubules also colocalized with the VPS35 subunit of the cargo-selective retromer complex (Fig. 7E). Endosomal F-actin and VPS35 are known to be involved with retromer-dependent recycling (Burd and Cullen, 2014). Using live-cell microscopy, we observed that that the transmembrane R-SNARE protein VAMP3 is sorted out through JIP4-positive tubules (Fig. 6F). We show several examples where VAMP3 exits from the macropinosome during a JIP4-tubulation event (Fig. 7F; Fig. S7A,B, Movie 4). Taken together, this indicates that JIP4 preferentially labels retromer-containing tubules, suggesting that it is involved in retromer-dependent trafficking.

## DISCUSSION

In this study, we described that a previously uncharacterized region of JIP4 interacts with the PH domain of the phosphoinositide-binding protein Phafin2, recruiting JIP4 to the membrane of early macropinosomes. Phafin2 binds PtdIns3P – generated by the PtdIns 3-kinase VPS34 – through its FYVE domain, which localizes it to endosomes and macropinosomes (Pedersen et al., 2012; Tang et al., 2017). Our data show that genetic ablation of Phafin2 or the removal of PtdIns3P disrupt the localization of JIP4 to macropinosomes. Recruitment of JIP4 by Phafin2 to membranes does not require other protein or lipid components found on macropinosomes, apart from the ones needed to anchor Phafin2 to the membrane. The JIP4 homolog JIP3 is not recruited by Phafin2, and the Phafin2 homolog Phafin1 is incapable of recruiting either JIP3 or JIP4. Consistent with this specificity of Phafin2 for JIP4, ablation of JIP4 did not interfere with the successful completion of macropinocytic internalization. JIP3, by contrast, has been reported to assist macropinosomes in moving through cortical actin (Williamson and Donaldson, 2019).

We found that JIP4 is enriched at subdomains of the macropinosome from which membrane tubules are generated. Moreover, down- or upregulation of JIP4 levels suppressed or promoted tubulation, respectively. JIP4-KO cells retained more of the fluid-phase marker dextran after macropinocytic uptake, and this increased cargo retention was found in both early (i.e. RAB5) and late macropinosome (i.e. LAMP1) compartments. In line with previous studies that have functionally implicated JIP4 in endocytic recycling (Montagnac et al., 2011), these JIP4-positive tubules contain transmembrane cargo (i.e. VAMP3), the retromer recycling complex (Burd and Cullen, 2014), and emanate from actin-enriched subdomains on the macropinosome (Burd and Cullen, 2014).

Whereas Phafin2 shows a biphasic localization to macropinosomes – to nascent macropinosomes and also to early macropinosomes (Schink et al., 2017 preprint) – JIP4 only binds to Phafin2 at the early macropinosome stage. This suggests that the interaction site between Phafin2 and JIP4 is inaccessible on nascent macropinosomes.

Additionally, we observed that this interaction is specific for JIP4 and Phafin2, as Phafin2 does not interact with JIP3 and Phafin1 does not bind to JIP4. This is important to note, since several other studies have previously proposed overlapping functions of JIP3 and JIP4, and some phenotypes had been reported under conditions of JIP3 and JIP4 double-KD, or JIP3 or JIP4KO (Boecker et al., 2021; Gowrishankar et al., 2021; Marchesin et al., 2015; Sato et al., 2015; Vilela et al., 2019; Williamson and Donaldson, 2019). In comparative structural and biochemical analysis, the similarity of the first two coiled-coil regions has been noticed (Isabet et al., 2009; Williamson and Donaldson, 2019). Our data showed that the Phafin2 recruitment mechanism distinguishes between the two isoforms. Likewise, despite the high sequence similarity between the PH domains of Phafin1 and Phafin2, only Phafin2 competently recruited JIP4. Interestingly, LYTL also appears to be JIP4 specific, as JIP3 could not be detected at lysosomes (Bonet-Ponce et al., 2020).

Our data do not exclude the possibility that other proteins – perhaps in combination with Phafin2 – may contribute to JIP4 localization. Indeed, the Phafin2-binding site on JIP4 is distinct from that for ARF6 (Isabet et al., 2009), motor proteins (Cockburn et al., 2018; Montagnac et al., 2009; Vilela et al., 2019), RAB36 (Matsui et al., 2012), and other RAB-binding motifs previously reported (Waschbüsch et al., 2020), raising the possibility of simultaneous interaction. Experiments regarding Phafin2 KO, truncation and chimeras reported in our study here, suggest that most of the tubule targeting of JIP4 is mediated by the Phafin2-binding activity, at least on PtdIns3P-positive membranes. JIP4 binding to ARF6 is not required to target tubules. In the absence of the Phafin2-binding activity, i.e. in the case of JIP3 PBR*, JIP4 does retain a miniscule amount of tubule targeting (see the cyan-marked macropinosome and the slight bump in the JIP4-J3PBR* curve in Fig. 5E,F). It is possible that a motif outside the PBR has some affinity to an as-yet-unidentified protein enriched at tubules, but such motif would also have to be specific for JIP4 and not shared with JIP3. An alternative possibility is that JIP3 PBR* has a very small residual affinity for Phafin2, but our *in vitro* and *in vivo* assays did not detect any such activity.

We found that JIP4 does not localize to the whole macropinosome membrane but, preferably, to tubules positive for the retromer component VPS35. This is in line with a previous study describing JIP4 localization to late endosomes in close proximity to the Wiskott-Aldrich syndrome protein and scar homolog (WASH), which organizes actin on retromer tubules, and that JIP3 and JIP4 are required for recycling of the matrix metalloprotease MT1-MMP via endosomal tubules (Marchesin et al., 2015). Based on the described binding of JIP4 to motor proteins, it is tempting to speculate that the tubular localization of JIP4 couples these membranes to the cytoskeleton and, thereby, drives tubule formation. Indeed, expression of Phafin2 in JIP4-KO cells did result in reduced tubulation, whereas expression of both Phafin2 and JIP4 strongly enhanced tubulation. Moreover, although binding of JIP4 to microtubule motors has been described (Balderhaar and Ungermann, 2013; Montagnac et al., 2009), it is less clear how the resultant forces would be transmitted to the membrane to cause tubulation. However, during LYTL, it has been reported that phosphorylated RAB10 physically interacts with JIP4 on the damaged lysosome membrane, recruiting JIP4 and driving tubulation (Bonet-Ponce et al., 2020). Our data indicates that Phafin2 performs a similar function, i.e. to couple JIP4 to the membrane of macropinosomes – and probably endosomes – in a PtdIns3P-dependent manner. Notably, the V416A and I421A point mutations that disrupt ARF6 binding (Isabet et al., 2009) did not affect tubule targeting, indicating that – at least for retromer-dependent recycling – ARF6 does not perform this mechanical role.

Whereas JIP4 has been proposed to play a role in macropinocytosis (Williamson and Donaldson, 2019), we did not observe any defects regarding macropinosome formation in cells deleted for JIP4. By contrast, we did observe enhanced intracellular levels of dextran in JIP4-KO and -KD cells after fluid-phase uptake, indicating that these cells retain more dextran within the cell. This is in line with our observation that Phafin2 is required in early steps of macropinocytosis, whereas JIP4 recruitment only occurs after macropinosomes have successfully entered the cell and have matured into early macropinosomes. The increased intracellular dextran levels are not explained by increased macropinocytosis, which we have tested and reported as unchanged in the present study, but possibly result from a slowdown of recycling from the internalized macropinosomes. An alternative possibility is that membrane and cargo are still removed from the original macropinosome but diverted to a trafficking route within the cell – but our data are insufficient to distinguish between these possibilities. However, we noticed that these results are consistent with those reported for *Dictyostelium discoideum*, where disruption of retromer-dependent recycling causes a decreased rate of recycling from macropinosomes to the plasma membrane (Buckley et al., 2016).

In conclusion, our data establish Phafin2 as a potent recruiter of JIP4 to PtdIns3P-positive membranes, where it promotes the formation of membrane tubules. These membrane tubules bear characteristic markers of retromer-dependent recycling zones. The removal of JIP4 does not impair macropinocytosis and promotes neither macropinocytosis nor macropinocytic uptake. Instead, cells that are lacking JIP4 contain more fluid-phase cargo, which is likely to result from a defect in endocytic recycling.

## MATERIALS AND METHODS

### Constructs, cells and culture conditions

Human retinal pigment epithelial (hTERT-RPE-1) cells (ATCC CRL-4000) were grown in DMEM/F12 medium (Gibco) with 10% fetal bovine serum (FBS), 5 U/ml penicillin and 50 µg/ml streptomycin. HeLa cells were grown in DMEM (Gibco) with 10% FBS, 5 U/ml penicillin and 50 µg/ml streptomycin. HT1080 cells (ATCC CCL-121) were grown in DMEM (Gibco) with 10% FBS, 5 U/ml penicillin and 50 µg/ml streptomycin. Cell lines were tested for mycoplasma contamination. Cell lines stably expressing constructs were generated by lentiviral transduction at low multiplicity of infection and subsequent antibiotic selection for integration of the expression cassette. The following antibiotics were used: puromycin (2.5–5 µg/ml), blasticidin (10 µg/ml), Geneticin (500 µg/ml). VSV-G pseudotyped lentiviral particles were packaged using a third-generation lentivirus system in Lenti-X cells. All lentiviral constructs except Phafin2 were expressed from a phospho-glycerate kinase (PGK) promoter. LAP-tag fusions of Phafin2 were expressed under control of the PGK promoter, whereas other tagged Phafin2 constructs were expressed from an elongation-factor-1α (EF1α) promoter. Transfections were carried out using Fugene 6 (Promega) at a ratio of 3 µl reagent per µg DNA. Halotag fusion proteins were labeled with Janelia Fluor 646 Halotag Ligand (Promega) for live-cell imaging, or with Janelia Fluor 549 Halotag Ligand (Promega) for CLEM. Constructs were generated using standard molecular biology techniques and, where appropriate, verified by Sanger sequencing.

### Generation of JIP4-KO cell lines

The guide (g)RNA sequence (5′-CCTGGACTCGGTGTTCGCGC-3′) was cloned into plasmid pX458 with GFP replaced with iRFP. The construct was nucleofected into hTERT-RPE-1 cells (Lonza) and sorted by flow cytometry into single cells grown on a 24-well plate. The resulting colonies were assayed by western blotting and sequencing of cloned PCR fragments from a genomic PCR flanking the predicted Cas9 cleavage site. The PCR primers for the genomic PCR were 5′-CTGGAGGACGGTGTGGTGTA-3′ and 5′-CGCTCGTACTGGGTGATGAG-3′, with a product length of 266 bp, which was cloned into pJet (ThermoFisher Scientific) for Sanger sequencing. Two cell lines that lacked expression of JIP4 were identified by western blotting. Genomic PCR followed by Sanger sequencing showed that one cell line had two genomic alterations. One of the two alleles had a deletion of one guanine at position 195, whereas the other allele had a deletion of one cytosine at position 196. Both mutations result in a −1 frameshift. Sequencing of the other cell line showed that both alleles had a insertion of one cytosine at position 195, resulting in a +1 frameshift. The cell line containing deletions in both alleles (G195, C196) – resulting in a frameshift – was chosen for subsequent use and further validated by immunofluorescence assay. Sanger sequencing chromatograms, western blot results and immunofluorescence images are shown in Fig. S3C–E.

### Antibodies and staining reagents

Antibodies and reagents used were anti-JIP4 (Cell Signaling, cat #5519, western blotting 1:1000, immunofluorescence 1:100 or 1:500; Fig. 2C). Anti-Phafin2 (Sigma-Aldrich HPA024829; 1:1000 western blotting); anti-GFP (Roche, cat #11814460001, western blotting 1:1000); anti-EEA1 antiserum (gift from Ban-Hock Toh of Monash University, Australia; immunofluorescence 1:160,000); anti-VPS35 (Abcam cat #ab10099, immunofluorescence 1:200) and phalloidin CF568 (Biotium Cat #00044, 1:200).

### Plasmids

JIP4 was obtained by PCR from cDNA reverse transcribed with Superscript IV (Life Technologies) prepared from RPE-1 cells. Various constructs of JIP3 were cloned from pEGFP-JIP3 (gift from Philippe Chavrier, Institute Curie, Paris, France). VAMP3 was cloned from pEGFP-VAMP3 (Addgene #42310), which was a gift from Thierry Galli (Galli et al., 1998). Coronin1B-mCherry (Addgene #27694) and Dynamin2-mCherry (Addgene #27689) were gifts from Christien Merrifield (Taylor et al., 2011). pX458 (Addgene #48138) was a gift from Feng Zhang (Ran et al., 2013). Other constructs were cloned using standard molecular biology techniques.

### RNA depletion

Silencer Select siRNAs against JIP4 (JIP4 siRNA #1: s17232 Sense Seq 5′-GAGUAGUUUAGAUAAGUUA-3′, JIP4 siRNA #2: s17233 Sense Seq 5′-GGAUCUGACGGGUGACAAA-3′) and non-targeting control siRNA (Silencer Select Negative Control No.1 siRNA Cat #4390843) were purchased from Ambion (Thermo Fisher Scientific). Cells were seeded in six-well plates at 30% confluence and transfected with 50 nM final siRNA concentration using Lipofectamine RNAiMax (Life Technologies) according to the manufacturers’ instructions. 20–24 h after transfection, medium was replaced with fresh medium. HT1080 cells were then used for experiments 48 h after knockdown (from when siRNA was added), whereas RPE-1 cells were replated onto new six-well plates at the 48 h time point and used for experiments at 72 h. All knockdowns were verified by western blotting (Fig. S4).

### Immunoprecipitation

hTERT-RPE-1 cells stably expressing GFP or GFP-JIP4 were grown in 6 cm dishes up to 80% confluence, washed once with phosphate buffered saline (PBS) and lysed in lysis buffer [25 mM HEPES pH 7.5, 100 mM NaCl, 1 mM DTT, 0.5% IGEPAL, 1× Complete protease inhibitor (Roche), 1× phosphatase inhibitor 2 (Merck) and 1× phosphatase inhibitor 3 (Merck)]. Cell debris was removed by pelleting at 5000 ***g*** for 10 min. GFP-Trap beads were added and gently mixed for 2 h at 4°C. Beads and supernatant were magnetically separated and beads were washed four times with lysis buffer before final denaturation with 1× Laemmli Buffer at 100°C for 20 min.

For tandem affinity purifications, hTERT-RPE-1 cells stably expressing LAP or LAP-Phafin2 were grown in 15 cm dishes up to 80% confluency. Cells were stimulated with hepatocyte growth factor (HGF) (Merck) at 50 ng/ml for 10 min before the experiment. Cells were lysed in lysis buffer (50 mM HEPES pH 7.5, 0.1% NP40, 150 mM KCl, 1 mM EGTA, 1 mM MgCl_2_, 1 mM DTT, 15% glycerol), cleared by centrifugation at 20,000 ***g*** for 20 min, and incubated with GFP-Trap beads for 2 h. Following four washes in lysis buffer, the GFP-Trap bead-bound fraction was incubated with recombinant TEV (Merck) overnight at 4°C. The supernatant fraction was collected and incubated with S-protein beads (Merck) for 2 h. Bound fractions were washed four times in lysis buffer and processed for mass spectrometry analysis.

For APEX2 proximity labeling proteomics, hTERT-RPE-1 cells stably expressing APEX2-mCitrine-Phafin2 fusions or control fusions were grown in 15 cm dishes to 80% confluency. Cells were incubated for 3 h in 500 µM Biotin-Phenol (Iris) at 37°C, washed in PBS and incubated for 2 min in 2 mM H_2_O_2_ (Merck) at room temperature, and subsequently washed four times in Quencher solution [5 mM Trolox (Merck), 10 mM Na-Ascorbate (Merck)]. Cells were lysed on ice in RIPA buffer (50 mM Tris HCl pH 7.5, 150 mM NaCl, 1% Triton X-100, 0.1% SDS, 0.5% NaDOC, 5 mM EDTA, 1 mM DTT) supplemented with protease inhibitors and 10 mM Na-Ascorbate, cleared by centrifugation at 20,000 ***g*** for 20 min, and passed through desalting columns to eliminate free biotin-phenol. Lysates were subsequently incubated for 2 h at 4°C with Streptavidin Dynabeads (Invitrogen M-280), and beads were successively washed with RIPA (twice), PBST (twice), 1% SDS (twice), 4 M Urea (twice), and PBS (five times) before being processed for mass spectrometry analysis.

### LC–MS/MS, protein identification and label-free quantification

Beads containing bound proteins were washed three times with PBS, reduced with 10 mM DTT for 1 h at 56°C followed by alkylation with 30 mM iodoacetamide in final volume of 100 µl for 1 h at room temperature. The samples were digested overnight with sequencing-grade trypsin (Promega) at 37°C, using 1.8 µg trypsin. Reaction was quenched by adding 1% trifluoracetic acid to the mixture. Peptides were cleaned for mass spectrometry using the STAGE-TIP method and a C18 resin disk (3 M Empore) (Rappsilber et al., 2003). All experiments were performed on a Dionex Ultimate 3000 nano-liquid chromatography (LC) system (Sunnyvale, CA, USA) connected to a quadrupole-Orbitrap (QExactive) mass spectrometer (ThermoElectron, Bremen, Germany) equipped with a nano-electrospray ion source (Proxeon/Thermo). For liquid chromatography separation we used an Acclaim PepMap 100 column (C18, 2 µm beads, 100 Å, 75 μm inner diameter) (Dionex, Sunnyvale, CA, USA) capillary of 25 cm bed length. The flow rate used was 0.3 μl/min, and the solvent gradient was 5–40% solvent B in 120 min, then 40–80% solvent B in 20 min. Solvent A was aqueous 2% acetonitrile in 0.1% formic acid, whereas solvent B was aqueous 90% acetonitrile in 0.1% formic acid.

The mass spectrometer was operated in the data-dependent mode to automatically switch between mass spectrometry (MS) and MS/MS acquisition. Survey full scan MS spectra (from m/z 300–1750) were acquired in the Orbitrap with a resolution (R)=70,000 at m/z 200, after accumulation to a target of 1,000,000 ions per quadruple. The method used allowed sequential isolation of the most-intense multiple-charged ions – up to ten, depending on signal intensity – for fragmentation on the higher energy C-trap dissociation (HCD) cell using high-energy collision dissociation at a target value of 100,000 charges or maximum acquisition time of 100 ms. MS/MS scans were collected at 17,500 resolution at the Orbitrap cell. Target ions already selected for MS/MS were dynamically excluded for 45 s. General MS conditions were: electrospray voltage, 2.0 kV; no sheath and auxiliary gas flow, heated capillary temperature of 250°C, heated column at 35°C, normalized HCD collision energy 25%. Ion selection threshold was set to 1e−5 counts. Isolation width of 3.0 Da was used.

MS raw files were submitted to MaxQuant software version 1.6.1.0 for protein identification (Cox and Mann, 2008). Parameters were set as follows: protein N-acetylation, methionine oxidation and pyroglutamate conversion of glutamic acid and glutamine as variable modifications. First search error window of 20 ppm and mains search error of 6 ppm. Trypsin without proline restriction enzyme option was used, with two allowed miscleavages. Minimal unique peptides were set to 1, and false-discovery rate (FDR) allowed was 0.01 (1%) for peptide and protein identification. Label-free quantification was set with a retention time alignment window of 3 min. The Uniprot human database was used (downloaded August 2013). Generation of reversed sequences was selected to assign FDR rates.

### Yeast two-hybrid and β-galactosidase assays

Yeast two-hybrid assays were carried out in the yeast strain L40 (ATCC MYA-3332), using LexA and Gal4-Activation Domain (GAD) as paired bait and prey N-terminal fusions (Brückner et al., 2009). The constructs were co-transformed into yeast and double-positive transfectants were selected using leucine+tryptophan drop-out agar medium. Several clones were picked of each condition and pooled to grow overnight liquid cultures for β-galactosidase assay. Liquid β-galactosidase assays were carried out by lysing yeast cells with 1 ml of lysis buffer (100 mM Tris HCl pH 7.5, 0.05% Triton X-100) and snap freeze/thaw in liquid nitrogen. Β-galactosidase activity was assayed by hydrolysis of ortho-nitrophenyl-β-galactoside to ortho-nitrophenol in reaction buffer (100 mM sodium phosphate buffer pH 7.0, 10 mM KCl, 1 mM MgSO_4_) at 37°C. The reaction was stopped by addition of a sodium carbonate buffer (250 mM final concentration) and immersion in ice as soon as a yellow color was seen. Ortho-nitrophenol product was quantitated by absorbance at 420 nm. The reaction rate was calculated by dividing the quantity of ortho-nitrophenol product (concentration in a constant volume) by the time elapsed for each reaction tube, and normalized against quantity of yeast cells (absorbance at 600 nm of raw lysate). All experiments were assayed in technical duplicates (the complete experiment from picking of colonies to assay was run in parallel as technical duplicate) and the means of the technical duplicates in each experiment are reported as an experiment datapoint. 3–4 separate experiments were carried out for each figure reported.

### Immunocytochemistry

For immunocytochemistry results reported in Fig. 2A,B and Fig. S3D, hTERT-RPE-1 cells of the indicated genotype were grown on glass coverslips. The cells were washed once with ice-cold PBS and pre-permeabilized for 5 min with PEM buffer (80 mM PIPES pH 6.8, 5 mM EGTA, 1 mM MgCl_2_) containing 0.05% saponin on ice. The cells were then fixed for 20 min on ice with 4% paraformaldehyde in PBS and stained with primary antibody at the listed concentration overnight at 4°C in PBS containing 0.05% saponin. Secondary antibody staining was carried out for 1 h at room temperature in PBS containing 0.05% saponin. Samples were mounted in Mowiol on glass slides.

For all other immunocytochemistry results, hTERT-RPE-1 cells of the indicated genotype were grown in glass-bottomed MatTek dishes (MatTek Life Sciences) and labeled for 20 min in culture medium with Halotag ligand. The culture medium was exchanged for live-cell imaging solution (Thermo Fisher Scientific) containing 50 ng/ml HGF to stimulate macropinocytosis for 15 min. Cells were fixed by addition of equal volume of room temperature 4% paraformaldehyde (final working concentration 2% PFA) in 0.1 M PHEM buffer (80 mM PIPES, 25 mM HEPES, 2 mM MgCl_2_, 10 mM EGTA pH 6.9) for 15 min and washed three times with PBS and once with PBS containing 0.02% saponin. Primary antibody staining was carried out overnight at 4°C in PBS containing 0.02% saponin. Secondary antibody staining was carried out for 1 h at room temperature in PBS containing 0.02% saponin. Phalloidin staining where indicated was added into the secondary antibody step at 1:200. Four washes of PBS containing 0.02% saponin was carried out after each antibody step. Cells were imaged as described for ‘Live-cell microscopy’ below, but at room temperature.

### Live-cell microscopy

Live-cell imaging was performed on a Deltavision OMX V4 microscope equipped with three PCO.edge sCMOS cameras, a solid-state light source and a laser-based autofocus. Cells were imaged in live-cell imaging buffer (Invitrogen) supplemented with 20 mM glucose, at 37°C. Environmental control was provided by a heated stage and an objective heater (20–20 Technologies). Images were deconvolved using softWoRx software and processed in ImageJ/FIJI.

### Quantifying endogenous JIP4 on EEA1 structures

Cells of the listed genotype were processed and fixed for immunocytochemistry. 15 fields of view of each condition were acquired (typically 1-3 cells per field of view) without changing acquisition parameters. EEA1 positive structures of at least five pixels were segmented from each image and the mean pixel intensity of each structure in the JIP4 channel was obtained. Each dataset was normalized by the mean of the entire experiment to control for staining and acquisition variation. Custom measurement and analysis script is available at GitHub.

### Quantification of JIP4 associated to tubules

For data reported in Fig. 4B, cells of the listed genotype that stably expressed the 2xFYVE^WDFY2^ probe and mNeonGreen-JIP4 were stimulated with 50 ng/ml HGF to trigger macropinocytosis and imaged live. Videos were taken for 5 min at intervals of 3 s. Tubules (membrane deformations that exceeded six pixels in length, 80 nm/pixel) that formed during that time period were marked in the 2xFYVE^WDFY2^ channel. The cytoplasmic background fluorescence for JIP4 of each cell was estimated by taking a 100×100 pixel square and measuring the mean fluorescence in the JIP4 channel. Each identified tubule was classified as JIP4 positive when the JIP4 fluorescence signal intensity was ≥50% above that of the background intensity determined above. Each cell was treated as a single biological data point (proportion of tubules JIP4 positive).

For all other data regarding JIP4 (and other markers) at tubules, cells of the listed genotype stably expressing the 2xFYVE^WDFY2^ probe were stimulated with 50 ng/ml HGF to trigger macropinocytosis and imaged live when indicated, Videos were taken for 5 min at intervals of 3 s (fixed imaging was acquired as reported above). A three-pixel wide line was manually drawn around regions of interest (ROIs) in ImageJ as shown in example images across the tubules and the fluorescence intensity recorded in each channel. All lineplot data were aligned using the highest 2xFYVE^WDFY2^ probe fluorescence to set the tubule position. The cytoplasmic background for each tubule was defined as the mean of the fluorescence excluding a 15-pixel window centered on the tubule position. Normalized fluorescence intensity as reported in figures was derived as the fold-change of the cytoplasmic background intensity. Custom measurements and analysis script are available at GitHub.

### Quantification of coefficient of variation

RPE-1 or RPE-1 JIP4 KO cells stably expressing Phafin2-mTurquoise2 were transfected 1 day before the experiment with either empty vector or mNeonGreen-JIP4. Cells were stimulated with HGF (50 ng/ml) and time-lapse images were captured. The image frame corresponding to 30 s after the start of imaging was extracted and used for further analysis. All macropinosomes >1 µm in diameter were included in the analysis. A three-pixel-wide line was manually drawn in ImageJ around each macropinosome, such that the entire circumference of the macropinosome was included. ImageJ reports the average gray value of the three-pixel thickness at each position along the line. These values from ImageJ were used to compute the coefficient of variation of Phafin2 intensity along the circumference of each macropinosome.

### Measurement of protein fluorescence intensities at the macropinosome membrane

Live-cell imaging was performed as described above for RPE-1 cells expressing specified proteins. HGF (50 ng/ml) was used to trigger macropinocytosis and timelapse videos were captured. Newly formed macropinosomes were identified in time-lapse movies and manually tracked by using Phafin2 or membrane markers as reference. For each time point, a region of their limiting membrane was marked as region of interest. Fluorescence intensity of a circular ROI (diameter of ten pixels) surrounding the marked region was quantified in all image channels and measurements were exported for further analysis. Custom measurement and analysis script is available at GitHub.

### Correlation analysis of coronin1B and dynamin-2

A ROI within the cell was drawn manually, including most of the macropinosomes, excluding the nucleus and membrane ruffles at the edge of the cell. Coloc2 plugin in ImageJ was used to compute the Pearson correlation coefficient as reported, using the ROI as a mask.

### Flow cytometry and dextran uptake

Cells were seeded in six-well plates at a density of 1×10^5^ the day before the experiment. Medium was replaced with pre-warmed medium containing 0.25 mg/ml dextran of the indicated mass (experiments with RPE-1 cells included 50 ng/ml HGF) and cells were incubated at 37°C for 30 min. After incubation, cells were washed quickly five times with pre-warmed medium, trypsinized and placed on ice after neutralization of trypsin. Flow cytometry was performed shortly after trypsinization with an LSRII flow cytometer (BD Biosciences). When EIPA was used as a macropinocytosis inhibitor, it was added at 100 µM to the growth medium 20 min before the start of the experiment and was present throughout the incubation with dextran.

### Dextran fluorescence assessed by microscope

Cells were seeded in glass-bottomed MatTek dishes and grown overnight. Before imaging, medium was replaced with pre-warmed medium containing 0.5 mg/ml dextran conjugated to Alexa Fluor 488 (10 kDa) and 50 ng/ml HGF. Cells were incubated at 37°C for 30 min. After the incubation, cells were quickly washed four times with pre-warmed medium, once with PBS and then fixed for 10 min at room temperature using 4% paraformaldehyde in PBS. Thereafter, cells were gently washed three times in PBS and the plasma membrane labeled with wheat germ agglutinin conjugated to Alexa Fluor 647 (Molecular Probes) at 5 µg/ml for 10 min in PBS. Cells were then washed twice with PBS, nuclei labeled with Hoechst dye 33342 (Molecular Probes), and imaging was performed in PBS. *Z*-stack images of 6 µm were acquired at an interval of 250 nm and deconvolved. One cell was measured per acquired field of view; the field of view was typically only large enough to fully fit one cell. For whole-cell dextran fluorescence measurements, *z*-stack images were projected using the sum of intensities. Cell outlines were manually traced in ImageJ using the plasma membrane marker as a guide. Background values (compensation for residual nonspecific dextran and imperfect deconvolution) were obtained from a 100×100 pixel square outside cells and subtracted from the fluorescence measured inside the cells. For organelle specific values, the image plane that was most in focus was extracted from the stack. Organelles of at least five pixels, with an approximate diffraction limit of 240 nm, were segmented using the listed organelle marker and the fluorescence measured. Reported values were computed per cell. Each experiment was normalized to the average of all data points of that experiment to account for acquisition parameters. The latter were held constant for all image stacks acquired per experiment. Custom measurement and analysis script for organelles is available at GitHub (https://github.com/koschink/JIP4).

### Correlative light and electron microscopy

For correlative light and electron microscopy (CLEM), cells were seeded on gridded MatTek glass bottom dishes the day before the experiment. Light-microscopy was carried out as specified under ‘Live-cell microscopy’ with time-lapse acquisition, while cells were stimulated with 50 ng/ml HGF. Directly after live-cell imaging, fixation was carried out using a final concentration of 2% glutaraldehyde in 0.1 M PHEM buffer (80 mM PIPES, 25 mM HEPES, 2 mM MgCl_2_, 10 mM EGTA pH 6.9) for 1 h and post-fixation was done using 1% OsO_4_ and 1.5% KFeCN in the same buffer for 1 h. Samples were further stained en bloc with 4% aqueous uranyl acetate for 1 h, dehydrated in a graded ethanol series and embedded with Epon-filled BEEM capsules (EMS; Polysciences, Inc., 00224) placed on top of the MatTek dish. After polymerization, blocks were trimmed down to the regions that had previously been identified by using the OMX microscope and were now imprinted on the Epon block. 200 nm sections were cut on an Ultracut UCT ultramicrotome (Leica, Germany) and collected on formvar-coated slot grids. Samples were imaged using a Thermo ScientificTM TalosTM F200C microscope equipped with a Ceta 16 M camera. Single-axes tilt-series for tomography were acquired between −60° and 60° tilt angles with 2° increments. Tomograms were computed in IMOD using weighted-back projection (Kremer et al., 1996). 3D modeling was performed by manual tracing of the macropinosome membrane in IMOD software version 4.9.3. Display of tomogram slices was also performed using IMOD software.

### Rapamycin recruitment

The mitochondrial anchor was constructed by fusing tandem FKBP12 FK506-binding domains to an N-terminal Tom70-derived mitochondrial targeting signal, with mTagBFP2 as localization marker. The FKBP-rapamycin-binding (FRB) domain of mTOR with a T2098L stabilization mutation and mNeonGreen was appended to the C-terminus of Phafin2. The mCherry-tagged JIP4 was not further modified. These three constructs were transfected into RPE-1 cells as previously described and images acquired using live time-lapse microscopy. A final working concentration of 10 µM of SAR-405 was used to dissociate Phafin2 from vesicles, and a final working concentration of 250 nM of rapamycin was used to recruit tagged Phafin2 to the mitochondrial anchor, added 5 min after treatment with SAR-405. Images were acquired before treatment, 5 min after treatment with SAR-405 and approximately 30 min after treatment with rapamycin. Intensity measurements were obtained by segmenting images using the mTagBFP2 mitochondrial marker in ImageJ.

### Quantification of macropinosome success rates, diameters and frequencies

For macropinosome success rates, RPE-1 cells of the specified genotype expressing mNeonGreen-2xFYVE as a PtdIns3P marker and Myrpalm-mCherry as a plasma membrane marker were seeded onto MatTek dishes and imaged live after triggering macropinocytosis with 50 ng/ml HGF. Macropinosome formation was tracked using the PM marker, and a vesicle was counted as successful when it had acquired PtdIns3P, indicating transition into an early endosome stage. Macropinosomes that did not successfully transition invariably fused again with the plasma membrane and then disappeared (as shown in Fig. 7E). For quantification of macropinosome diameters and frequencies, RPE-1 cells of the specified genotype that expressed mNeonGreen-2xFYVE as a PtdIns3P marker were seeded onto MatTek dishes and imaged live after triggering macropinocytosis with 50 ng/ml HGF. Time-lapse videos were first segmented in ImageJ using the 2xFYVE marker to mark 2xFYVE-positive structures as ROIs. The ImageJ plugin Trackmate with Sparse LAP-Tracker was then used to track each structure over time. These tracks were output to be further processed using Python. Structures with a radius <400 nm, and structures that existed from the beginning of the time-lapse were excluded, so only newly formed macropinosomes were tracked. From this, the diameters and frequencies of newly formed macropinosomes were obtained. Custom measurements and analysis scripts are available at GitHub.

### Sequence alignments and visualization

Sequences were imported into Jalview2 (Waterhouse et al., 2009) and aligned using Clustal Omega (Madeira et al., 2019; Sievers et al., 2011). Color scheme was set in Jalview2 according to the Clustal X system to group aa by their physicochemical properties.

### Statistical analysis

Statistical analysis was carried out in Graphpad Prism (Graphpad Software). Student's *t*-test was used to compare two groups. ANOVA was used to compare multiple groups and Holm–Sidak was used to correct for multiple comparisons. The threshold for significance was set at *P*=0.05. All comparisons made are reported – regardless of significance. Comparisons in the figures are indicated as n.s. *P*>0.05, *P<0.05, **P<0.01, ***P<0.001.

## Supplementary Material

Supplementary information

Reviewer comments
