# Supplementary material for: JIP4 is recruited by the phosphoinositide-binding protein Phafin2 to promote recycling tubules on macropinosomes
Source: J Cell Sci. 2021 Jul 20;134(14):jcs258495. doi: 10.1242/jcs.258495 (PMC8325962; doi:10.1242/jcs.258495)
Supplement: Supplementary information [file joces-134-258495-s1.pdf]

A

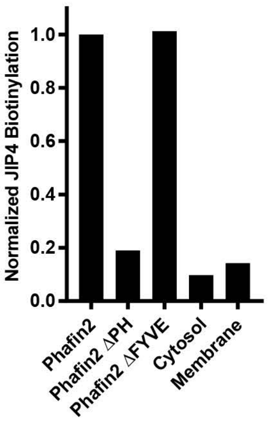

B

	Enrichment	Unique Peptides	Coverage [%]
Phafin2	67538	20	71.9
JIP4	28	22	24.1

C D

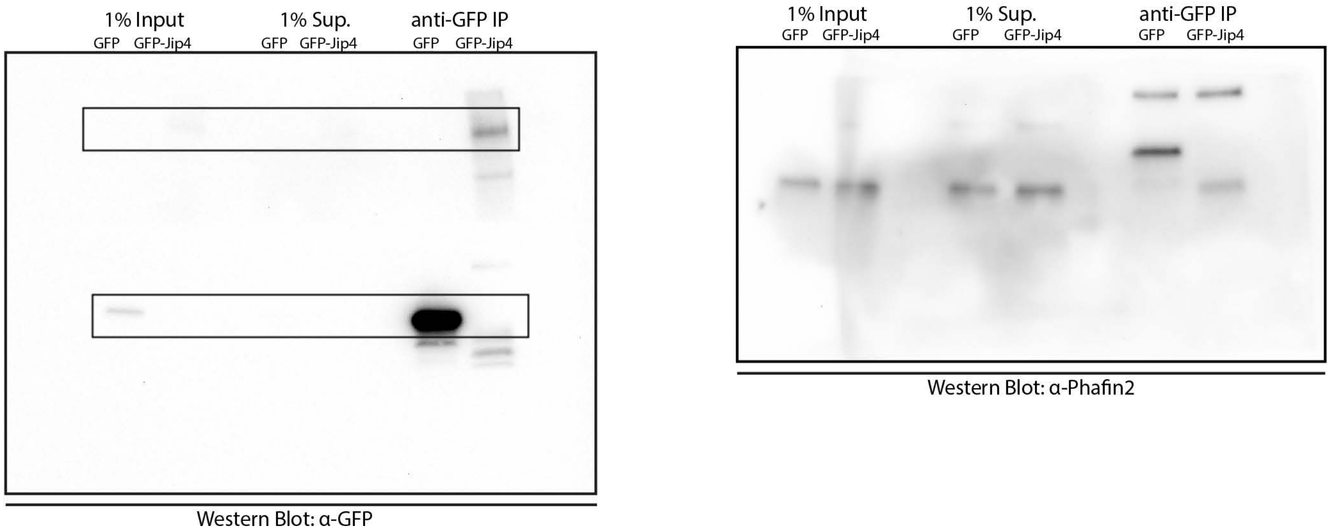

Fig. S1. JIP4 and Phafin2 interact in vitro. A) Biotinylated JIP4 detected in mass spectrometry following labeling with the specified APEX2 fusion constructs, normalized to wildtype Phafin2. Cytosol is a control consisting only of the soluble APEX2, while Membrane is a control consisting of the APEX2 fused to a signal peptide that targets it primarily to the plasma membrane. B) Endogenous JIP4 detected in mass spectrometry following affinity purification of tagged Phafin2, fold change over control cells expressing only the affinity tag. C) Uncropped source blot for Figure 1D, Phafin2. D) Uncropped source blot for Figure 1D, GFP.

Journal of Cell Science • Supplementary information

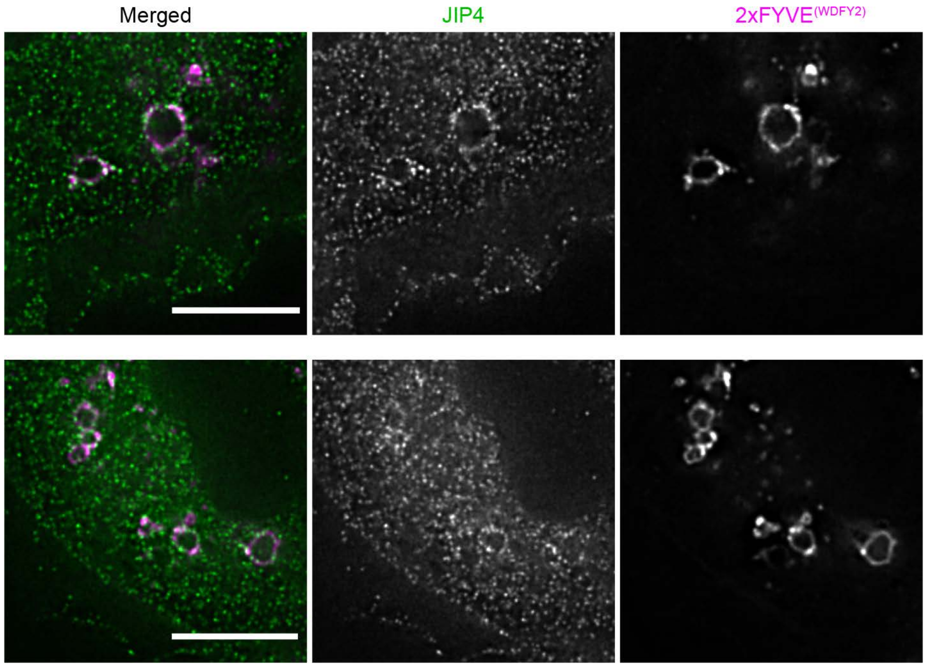

Fig. S2. Endogenous JIP4 localizes to PtdIns3P-positive endosomes. Representative images of RPE1 cells expressing 2xFYVE$^{(WDFY2)}$ to mark PtdIns3P-positive membranes, fixed without prepermeabilization, and immunostained against JIP4.

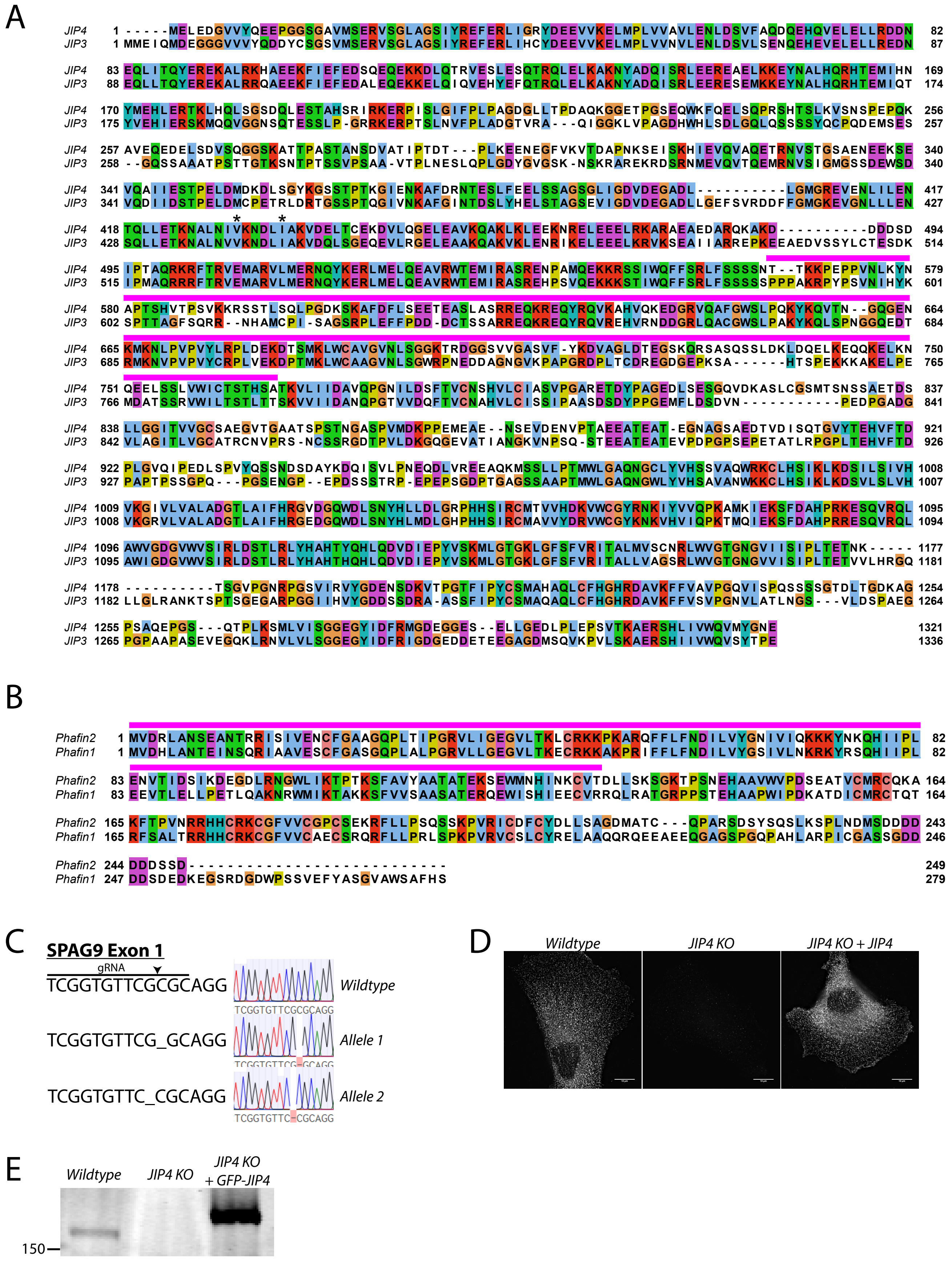

Fig. S3. Sequence alignments of JIP4 and Phafin2. Colour schemes according to Clustal X. A) Full length sequence alignment of human JIP4 and JIP3. The Phafin2-Binding-Region is marked in magenta, and the V416 and I421 positions are marked with an asterisk. Amino acid numbers differ slightly from Uniprot canonical isoform (shown here), but are identical to those reported in Isabet et. al. 2009. Exon 2 is spliced out in the construct we retrieved from RPE1 cDNA, and also presumably in Isabet et. al. 2009. B) Full length sequence alignment of human Phafin2 and Phafin1. The PH domain is marked in magenta. C) Guide RNA for CRISPR/Cas9 knockout. The predicted cut site is indicated. Sanger sequencing chromatograms show different frameshift insertions for both alleles. No wildtype sequencing results were recovered from the JIP4 KO cell line. D) Immunofluorescence using anti-JIP4. Images were acquired at the same settings and presented with equal brightness scaling. E) Western blot using anti-JIP4 on cell lysate from wildtype, JIP4 KO, and JIP4 KO expressing GFP-JIP4.

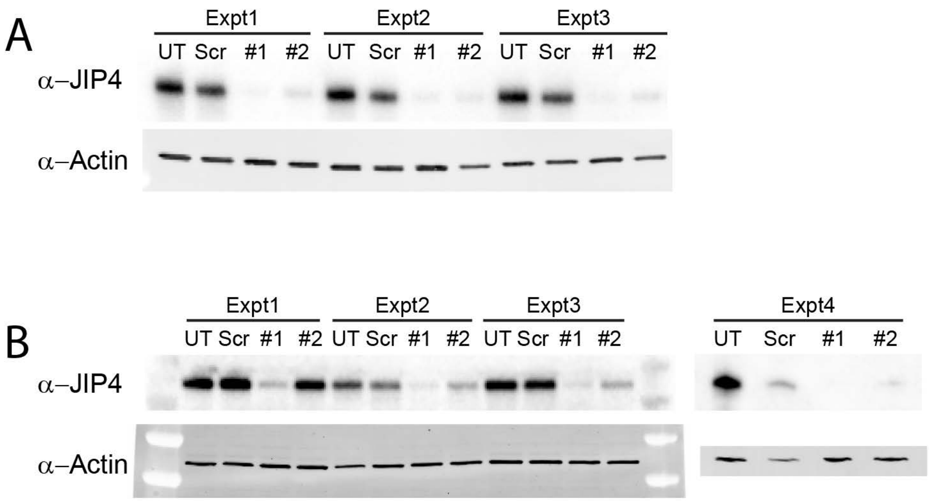

Fig. S4. JIP4 knockdown blots. A) Western blot against JIP4 and actin as a loading control for experiments reported in Figure 4F. B) Western blot against JIP4 and actin as loading control for experiments reported in Figure 4G.

Journal of Cell Science • Supplementary information

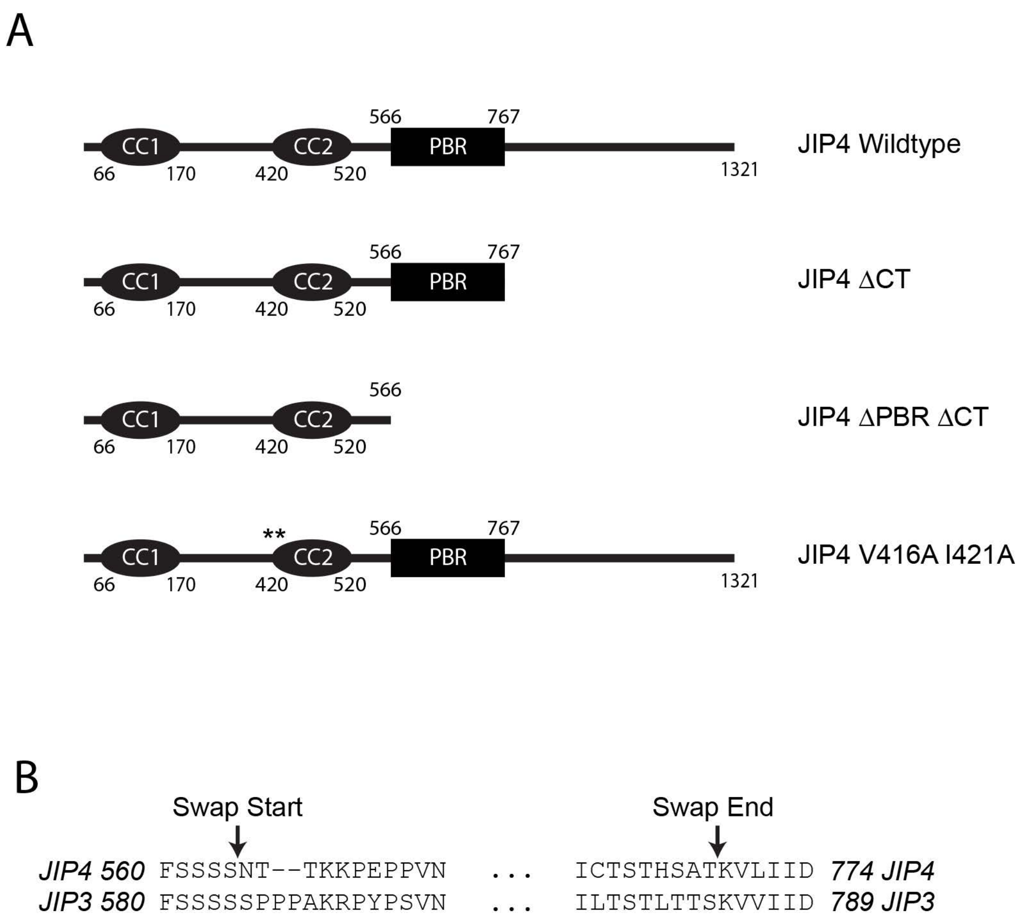

Fig. S5. Truncation and mutant constructs of JIP4 and JIP3. A) Schematic representation of JIP4 truncations and mutants used in Figure 5C, D. B) Swapping points for chimeric constructs used in Figure 5E, F.

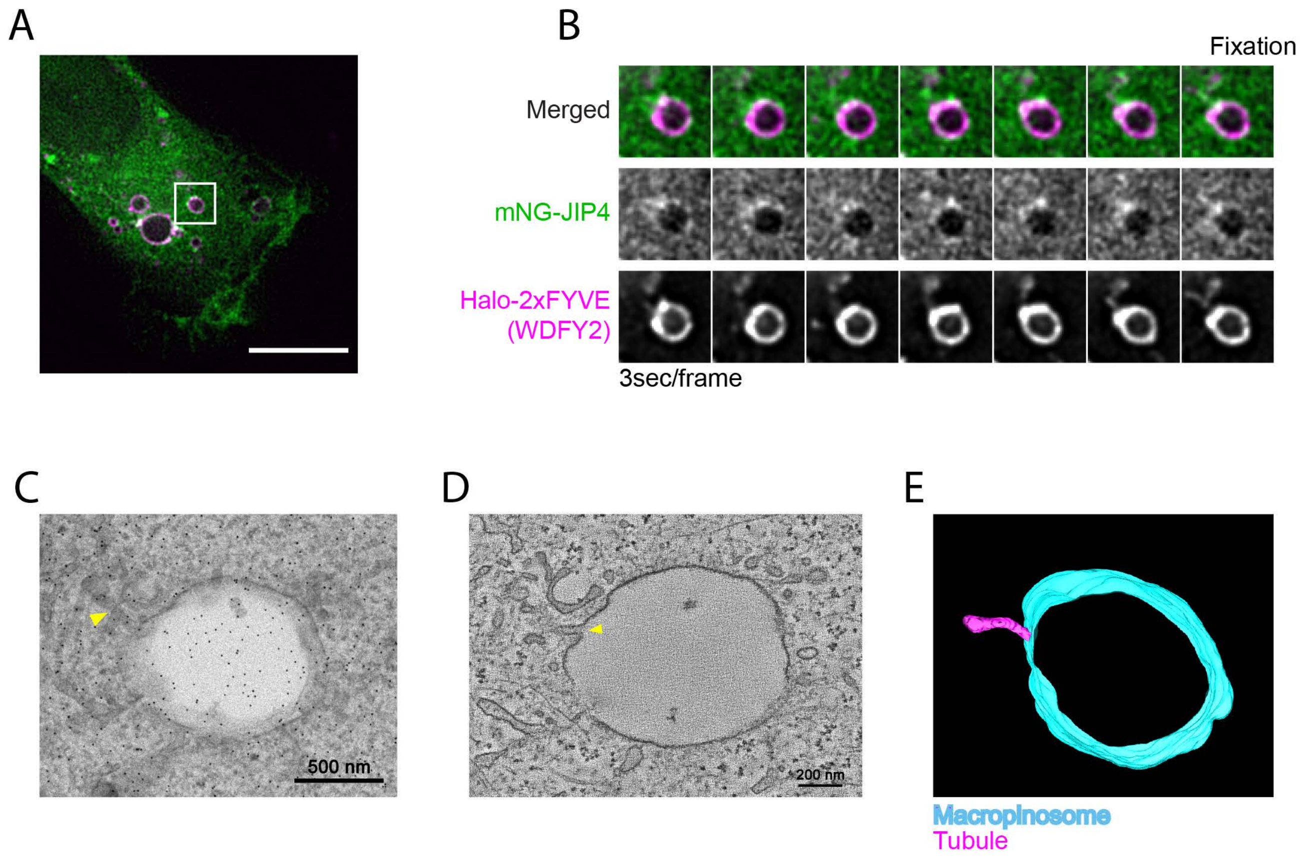

Fig. S6. Correlative light and electron microscopy of JIP4. Independently prepared CLEM specimen from that shown in Figure 6A-D. A) RPE1 cell expressing mNG-JIP4 and 2xFYVE$^{(WDFY2)}$. B) Timelapse montage of the macropinosome in C. C) Reconstructed electron micro-graph of macropinosome, yellow arrowhead marks tubule modeled in (E). D) Single slice through tomo-gram, yellow arrowhead marks the open neck of the tubule. E) Modeling of macropinosome membrane of C in cyan, tubule shown in magenta.

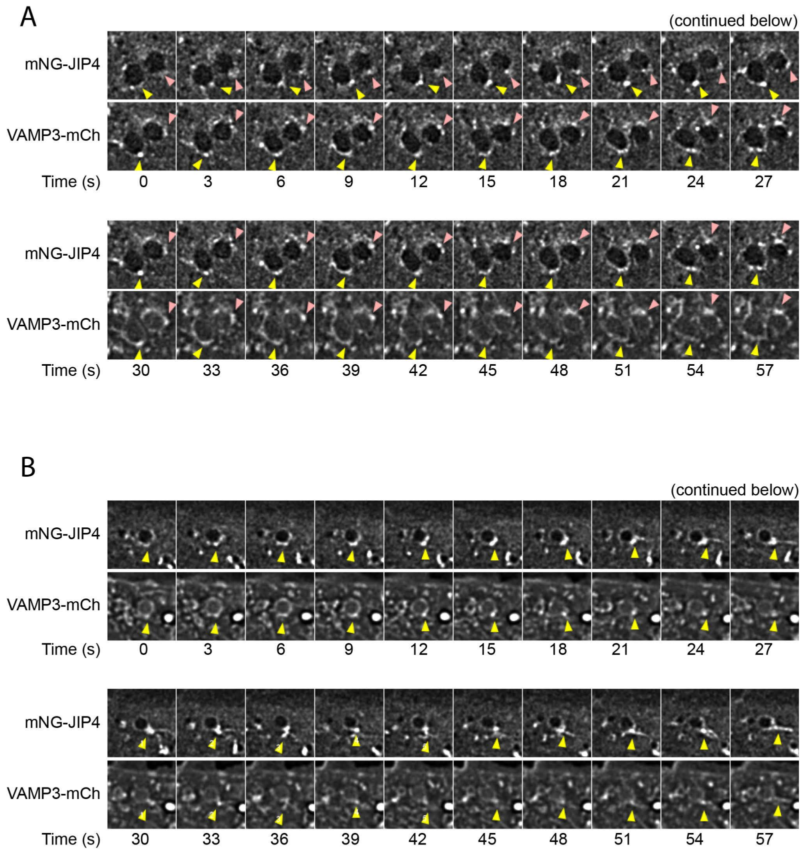

Fig. S7. VAMP3 is trafficked from the macropinosome in JIP4-positive structures. A) Montage of VAMP3 exiting the macropinosome from a JIP4-positive structure. B) Montage of VAMP3 exiting the macropinosome from a JIP4-positive structure. Montages shown here, in Figure 7, and in Movie 4 are from different cells.

Table S1. Yeast two-hybrid hits for Phafin2 against a leukocyte cDNA library.

Click here to download Table S1

Table S2. Mass spectrometry hits of biotinylated proteins using APEX2-tagged Phafin2 truncation mutants.

Click here to download Table S2

Table S3. Mass spectrometry hits of proteins after dual affinity purification of tagged Phafin2.

Click here to download Table S3

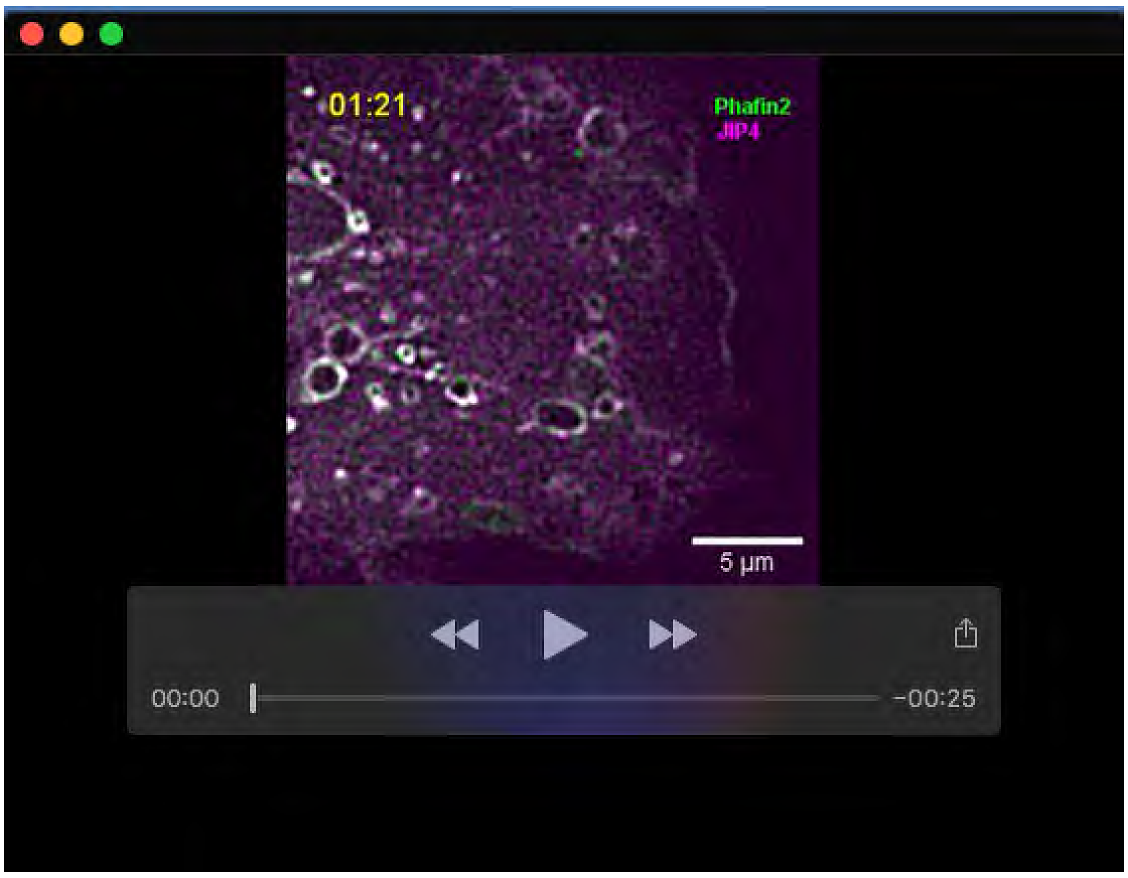

Movie 1. JIP4 localizes to Phafin2 positive early macropinosomes. Shown is a macropinocytosing RPE1 cell with Phafin2-mTurquoise2 (pseudocolored green) and mNeonGreen-JIP4 (pseudocolored magenta). Note that the nascent macropinosomes entering on the right display a burst of Phafin2 that is not accompanied by JIP4, while the early macropinosomes acquire both Phafin2 and JIP4.

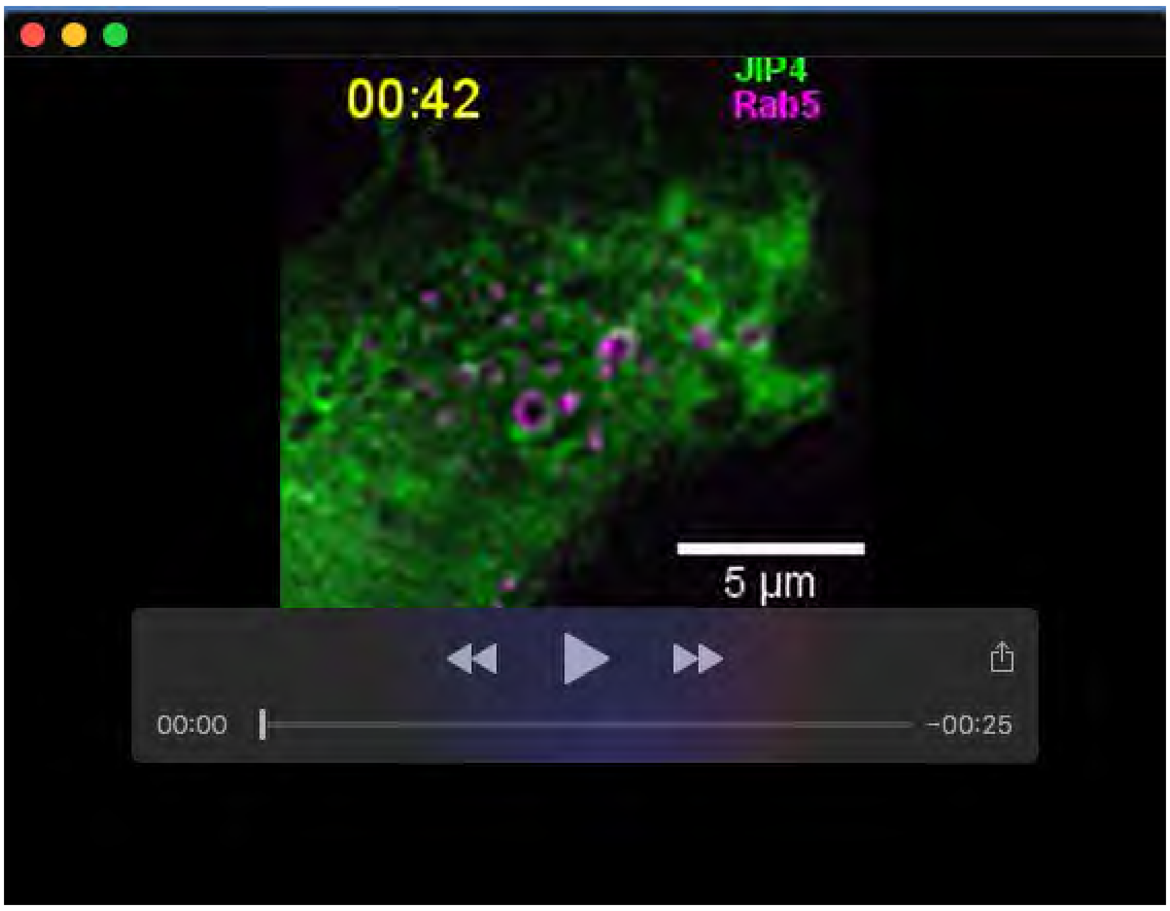

Movie 2. JIP4 localizes in subdomains on RAB5 positive macropinosomes. Shown is a macropino-cytosing RPE1 cell with mNeonGreen-JIP4 (pseudocolored green) and mCherry-Rab5 (pseudocolored magenta). JIP4 localizes to dynamic subdomains as the macropinosome acquires RAB5.

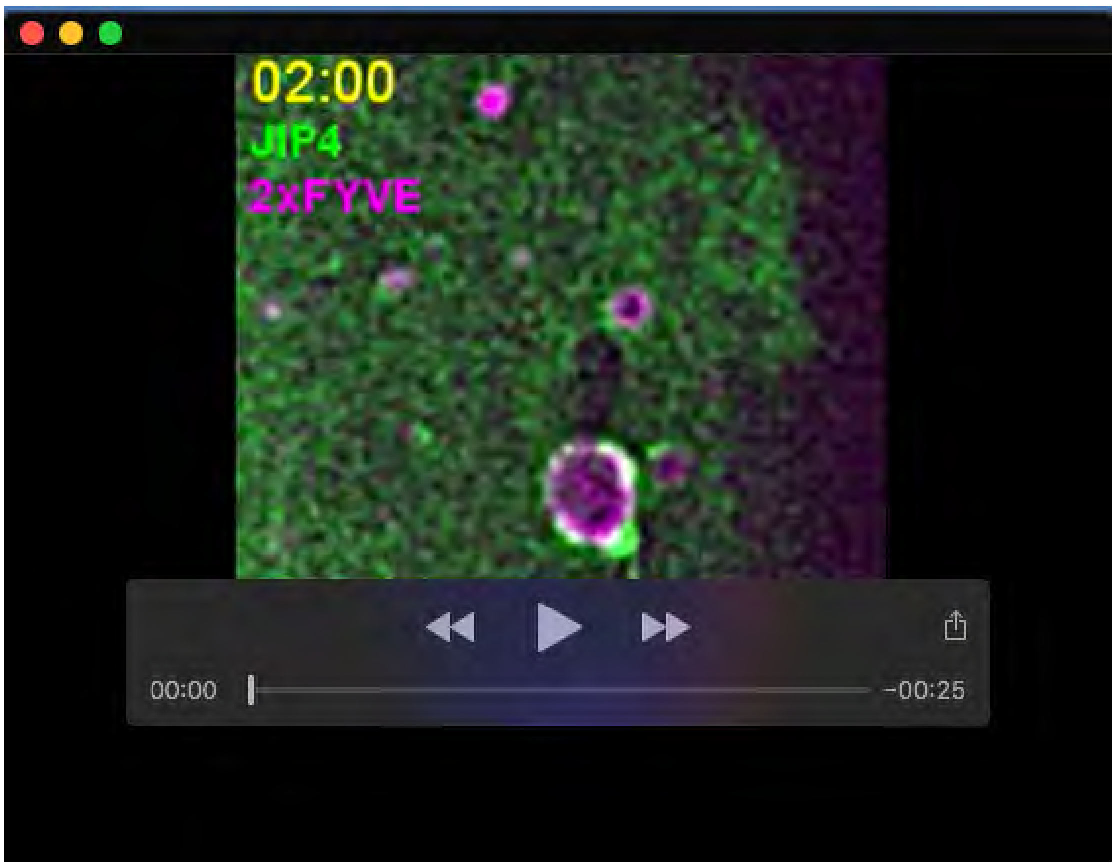

Movie 3. JIP4 preferentially localizes to PtdIns3P-positive tubules from macropinosomes. Shown is a macropinocytosing RPE1 cell with mNeonGreen-JIP4 (pseudocolored green) and Halo-2xFYVE(WDFY2) as a probe for PtdIns3P (pseudocolored magenta). Macropinosomes acquire both JIP4 and PtdIns3P, and JIP4 concentrates where PtdIns3P-positive tubules emerge and shortly thereafter.

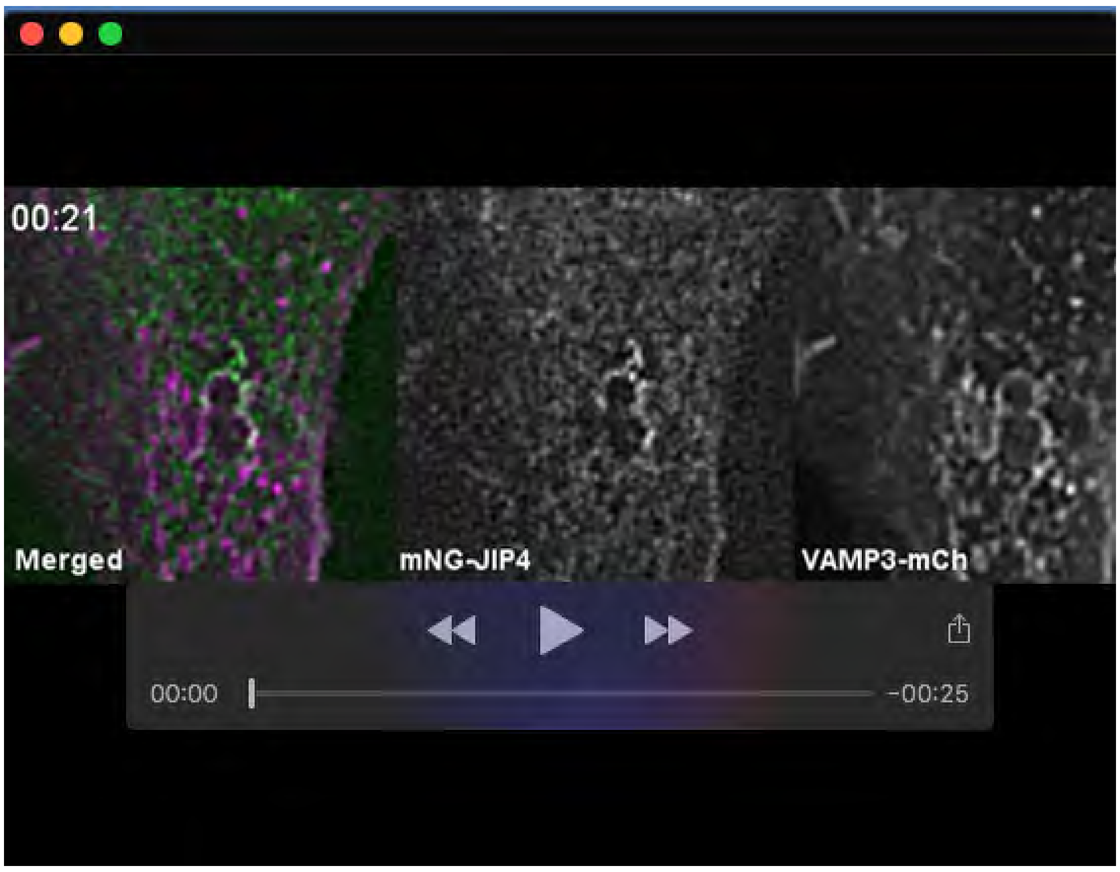

Movie 4. VAMP3 exits the macropinosome in JIP4-positive tubules. Shown is a macropinocytosing RPE1 cell with mNeonGreen-JIP4 (pseudocolored green) and VAMP3-mCherry (pseudocolored magenta). VAMP3 leaves the macropinosome in tubulating membrane structures that are positive for JIP4. The top macropinosome appears to deplete of VAMP3.
