# Supplementary material for: JIP4 is recruited by the phosphoinositide-binding protein Phafin2 to promote recycling tubules on macropinosomes
Source: J Cell Sci. 2021 Jul 20;134(14):jcs258495. doi: 10.1242/jcs.258495 (PMC8325962; doi:10.1242/jcs.258495)
Supplement: Reviewer comments [file joces-134-258495_review_history.pdf]

JIP4 is recruited by the phosphoinositide-binding protein Phafin2 to promote recycling tubules on macropinosomes

Kia Wee Tan, Viola Nähse, Coen Campsteijn, Andreas Brech, Kay Oliver Schink and Harald Stenmark
DOI: 10.1242/jcs.258495

Editor: David Stephens

Review timeline

Submission to Review Commons:	1 October 2020
Submission to Journal of Cell Science:	29 January 2021
Editorial decision:	4 February 2021
First revision received:	23 April 2021
Accepted:	27 May 2021

Reviewer 1

Evidence, reproducibility and clarity

In this manuscript, the authors showed that JIP4 and Phafin2 are recruited to vesicular structures and interact with each. Knocking out of Phafin2 decreased the vesicular localization of JIp4. The authors further showed that JIp4 is recruited to tubular structures by Phafin2 and potentially regulates the recycling of macropinosomes. Overexpression of JIp4 promoted the tubulation of macropinosomes, whilte knocking out of Jip4 inhibited the endosome tubulation and retained the macropinosome cargoes in the endolysosomal pathway. Although there are a few experiments and quantification need to be improved to better support the conclusions, overall the data presented in this manuscript is convincing.

The major technical weakness of this manuscript is that most of the experiments in this study were based on observation made with over-expression of tagged plasmids. The overexpression system could profoundly affect the localization of the target protein. One example is with the GFP tagged JIp3 in figure 4, where the authors showed a diffused pattern and it's unclear whether it represents the normal localization of JIp3 or it was an overexpression artefact. The only staining of endogenous JIP4 is in figure 6 A, and the image presented did not support the tubule localization of JIP4. Moreover, all the observations were made in RPE-1 cell line. The authors should at least perform experiments to look into whether this interaction is conserved in different cell types.

Specific points are listed as below

1. Figure 1 B-D, it is unclear how was the quantification performed. What is the replication number? Please include statistics analysis.

2. Figure 1 F, please include input blots for JIP4. Please compare the pull down of Phafin2 with JIP4 and JIP3.

3. Figure 1 G and I do not support that Phafin2 recruit JIP4. It seems that Phafin2 and JIP4 re recruited simultaneously by other protein. Please discuss.

4. Figure 1 J only quantified 4 macropinosomes. Please include more macropinosomes for the quantification.

© 2021. Published by The Company of Biologists under the terms of the Creative Commons Attribution License (https://creativecommons.org/licenses/by/4.0/).

5. Please analyze and discuss what phospholipid (PI(3,4,5)P3 or PI(3,4)P2 or PI(4,5)P2) does the phafin2 PH domain and interact with. Please analyze and discuss whether JIP4 is recruited to PI(3,4,5)P3 or PI3P.

6. Figure 2 B, it is unclear how did the authors performed the quantification. How did the authors determine the JIP4 signal "inside EEA1 positive vesicles"?

7. Figure 3 A, please label the plot to better indicate what are the x and y axis showing.

8. Figure 3 b, please include sequence alignment of the JIP3/4.

9. Jip3 seemed to be largely diffused even in WT RPE1 cells. This brings the question that whether the GFP-JIp3 overexpression altered the localization of Jip3.

10. Figure 4, the authors should provide localization evidence to show the JIP4 positive vesicles colocalize with dextran to prove that JIp4 is indeed recruited to macropinosomes. The authors should also label clathrin dependent vesicles and show that JIP4 do not colocalize with clathrin dependent vesicles.

11. The tubule localization of phafin2 and JIp4 presented in figure 4 C is not convincing. How did the authors distinguish between the tubules and a smaller vesicle adjacent to a large vesicle? Please clarify.

12. Please co-express JIp4 and Phafin2 and show the relative tubule localization of both proteins.

13. Figure 6, please quantify the co-localization of JIp4 and the indicated markers of recycling endosomes.

14. Figure 7, please provide high-content image for dextran uptake assay.

15. Figure 8, it is unclear whether the altered dextran "retention" is caused by altered recycling or degradation. The authors should perform experiments to probe these two possibilities.

Minor

Please label Figure 2 D.

Significance

The role of JIP4 in endosome tubulation is well documented. It has been shown that ARF6 recruits JIP4 on macropinosomes and regulates the tubule formation. Although the authors discovered another mechanism by which JIP4 might be recruited to macropinosomes, they did not show any major novel functions of JIP4. (PMID 30969891, 26504170)

This research would be interested to researchers focusing on endosomal trafficking pathways. One concern is that the authors used RPE-1 cells which has limited physiological relevance. To draw broader interests from general audience, the authors should prove that the proposed mechanism remain conserved in other cell types as well as primary cells.

© 2021. Published by The Company of Biologists under the terms of the Creative Commons Attribution License (https://creativecommons.org/licenses/by/4.0/).

Our lab has published multiple papers on endosomal trafficking. Similar analysis performed in this manuscript is used routinely in our lab.

Reviewer 2

Evidence, reproducibility and clarity

Summary

In this study, Wee Tan and colleagues report that a coiled-coil-containing protein JIP4 is targeted to macropinosomes by the PI3P-binding protein Phafin2 to promote tubulation and recycling from these organelles. The evidence presented in this manuscript, particularly with respect to imaging, is of high quality. The immediate conclusions are supported by the data, which is convincing and appears reproducible. It must be noted that use of SEM in place of SD for biological error determination is not appreciated, as it underplays the actual variation between cells and samples, particularly in imaging studies. Western Blot analysis is not of the highest quality (and lacks quantification), but is well supported the imaging studies.

Major points of discussion and criticism:

1. Line 85: 'This was interesting since JIP4 and it homologue JIP3 have been implicated in macropinocytosis (ref 17), although their function has remained largely unknown.' I was expecting that the authors would go on to determine the function of JIP4 in this process. However, given the limited mechanistic insights underpinning the manuscript, particularly considering the prior findings reported in refs #17 and #26, the present manuscript falls short in this regard.

2. Line 99: 'and that the local membrane environment is not required'. While it is clear from the data provided that the local membrane environment doesn't contribute to the interaction in question, it most likely contributes to the function of the resulting complex. In my view investigating this membrane context using the techniques already described in the manuscript, such as proximity-based biotinylation, would open the doors to understanding the mechanism through which this protein pair achieves tubulation and recycling of plasma membrane components in this vesicular pathway.

3. Line 113: My understanding is that the authors define macropinosomes based on their size and appearance. Given the wide variety of vesicular structures populating the endolysosomal system, its seems that the use of unique/canonical markers of macropinosomes should be implemented at least in some key experiments, such as those in Figure 1.

4. Line 150: 'This suggests that their interaction is specific.' The authors present compelling evidence that JIP4 and Phafin2 make a unique pairing with respect to their homologues. However, considering that the interaction between these proteins forms the central focus of the manuscript, it is suggested that the authors take this opportunity to more closely define the nature of this interaction based on the sequence similarities/difference amongst the homologues.

5. Line 217, Fig. 7G-I: The authors' findings regarding increased intracellular Dextran levels do not correspond with data presented in ref #17 in what I understand to be largely the same experiment. Is this a cell-type specific effect, or is there an alternate explanation?

6. No working model is presented to summarise the key findings of the study.

Minor points:

D is missing in Figure 2

Some form of quantification should be provided in figure 3E and F

Supplementary Fig. 1 has display panels A-D, which are not matched by the legend.

© 2021. Published by The Company of Biologists under the terms of the Creative Commons Attribution License (https://creativecommons.org/licenses/by/4.0/).

Significance

The manuscript is rather observational in nature. The scope of the study is rather limited, considering that it connects 2 proteins already known to function within the endocytic pathway. In its present form, the study neither identifies new endocytic players nor elaborates on the surrounding molecular context pertaining to the collaboration between JIP4 and Phafin2 in micropinocytosis. Taking this together with prior literature on JIP4 in macropinocytosis and endocytic recycling (references #17 and 26), the present findings do not present substantial conceptual or mechanistic advances in my view. I would recommend that the authors pursue new mechanistic insights, as noted under Major point #2, and evaluate consequences of JIP4 inactivity on the abundance/function of cell surface proteins subject to the macropinocytosis pathway to bolster the impact of their study.

Reviewer 3

Evidence, reproducibility and clarity

Summary

In the manuscript entitled "JIP4 is recruited by the phosphoinositide-binding protein Phafin2 to promote recycling tubules on macropinosome", Kia Wee Tan and colleagues show that the adaptor protein JIP4 physically interacts with the PI3P-binding protein Phafin2. The authors indicated the sites of interaction between Phafin2 and JIP4 as sites on macropinosomes where recycling tubules originate. This interaction is required for JIP4 localization on macropinosomes and is specific for JIP4, since its homologue JIP3 does not bind to Phafin2 and is not recruited to macropinosomes. In addition, they show that PI3P is required for the localization of Phafin2 and hence of JIP4 on macropinosomes. The authors also generated a JIP4-KO cell line that they used with live and fixed samples to perform functional studies on JIP4 that led the authors to the conclusion that JIP4 is involved in recycling from macropinosomes, since in the absence of JIP4 the cargo they used to track macropinosomes (10KD dextran) is retained within intracellular organelles.

Major comments:

The authors build their manuscript on JIP4 on their previous observation that Phafin2 is a novel regulator of macropinosome formation (ref 16.) However, this observation is only available at bioRxiv as an unreviewed manuscript since 2017 (Schink, K.O., et al., The PtdIns3P-binding protein Phafin2 escorts macropinosomes through the cortical actin cytoskeleton. 2017, bioRxiv) Is there a more updated or different and reviewed version of these data?

While the interaction of JIP4 and Phafin2 is nicely studied and reported, the major problem I see concerns the conclusion that JIP4 localizes on tubules and promotes tubulation and recycling from macropinosomes.
In fact, tubules are not clearly visible in any of the images reported in the Figures. These are visible only for some of the JIP4-positive structures in suppl. Video1. Instead, the figure images clearly show that the colocalization of JIP4 with macropinosomes is restricted to specific domains. In many instances it is difficult to discriminate whether these domains pertain to the macropinosome surface or are instead endosomes tethered to/fusing with macropinosomes. The authors should provide much more convincing evidence of JIP4 association with/effect on tubulation at macropinosomes.

The functional studies on JIP4-KO cells do not result in any conclusive evidence since a block of macropinosomal recycling should lead to an increase in macropinosome area, which is not reported among the phenotypes induced by JIP4-KO. The authors instead describe that the cargo they use to trace macropinocytosis, i.e. 10KD dextran, accumulates in JIP4-KO cells 30 min after internalization. Here there are three points the authors should/could consider:

© 2021. Published by The Company of Biologists under the terms of the Creative Commons Attribution License (https://creativecommons.org/licenses/by/4.0/).

1. The accumulation of dextran occurs in compartments that do not appear, judging by the images, to be macropinosomes but rather endosomal structures. Higher resolution images corroborated by morphometric analysis are necessary

2. 70KD dextran (internalized exclusively by macropinocytosis) would be preferable to 10KD dextran since the latter can be internalized also by fluid phase and clathrin-mediated endocytosis. The use of EIPA to discriminate macropinocytosis from endocytosis is questionable since EIPA inhibits other forms of internalization under certain conditions (Canton J, Front Immunol 9: 2286) . In addition the authors do not show the impact of JIP4 depletion on macropinocytosis (as the number of macropinosomes/cell), and they do not clearly demonstrate that the higher amount of intracellular dextran is due to defective recycling rather than increased internalization. Furthermore, given the expertise of the authors with live imaging, the visualization of JIP4-positive tubules carrying the cargo (dextran) would add strong evidence that the JIP4-positive tubules are actually used for the recycling of cargos. For example, dextran recycling and release by WT and JIP4-KO cells could also be measured by TIRF microscopy. In addition, the functional role of JIP4 in cargo recycling from early macropinosomes has been tested in WT and JIP4-KO cell lines. In this case it would be beneficial for the claim and for the specificity of JIP4-mediated cargo recycling to perform a rescue experiment in JIP4-KO cells by overexpressing JIP4.

3. They should at the same time follow an endocytic tracer to exclude an impairment in endocytic (and not specific macropinocytic) recycling.

Although the authors show by different means the physical and functional interaction of Phafin2 and JIP4, most of the data lack quantitative analysis and statistical evaluation of the results (see Fig.3E, F; 4A, B; Fig.5 and Fig.6 and see minor comments).
In most cases they show colocalization of JIP4 with other markers by plotting the fluorescence intensities of the two proteins in a Region of Interest without providing any quantitative or statistical analysis, the results shown in Fig.3E, F; 4A, B; Fig.5 and Fig.6 cannot be more than observations.

A general consideration regards the use of a single cell line (RPE1) and a single JIP4-KO line (RPE-JIP4-KO) that raises concerns about how general (or cell-specific) are the claims proposed in the manuscript. Testing the model described in the manuscript in a different cell line and, at least, in two other JIP4-KO clones (or in RPE1 treated with siRNAs against JIP4) would add substantial strength to the manuscript. The suggested supplemental experiments are fully in line with the expertise of the authors and with the methods already applied in the manuscript and can be completed in 3-6 months.

Minor comments:

Fig. 1B-C-D: error bars and statistical analysis should be added.

Fig. 1E: Number of replicates and statistical analysis has to be added.

Fig. 1F: the authors should include the WB with anti-GFP or anti-JIP4 to show immunoprecipitation efficiency of the bait.

Fig. 2C: The use of Rapamycin at 250nM for 30 minutes can reduce mTOR activity and thus induce autophagy. To exclude any interference of the mTOR/autophagic pathway, the authors should test Rapamycin at lower concentration/shorter time of treatment or include in the manuscript (as supplemental data) the evaluation of mTOR activity by measuring phosphorylation of S6-Kinase in RPE either untreated and treated with 250nM Rapamycin.

Fig. 3C-D: error bars and statistical analysis should be added.

Fig. 3E-F: Number of replicates, quantitative and statistical analysis has to be added. Fig. 3F: scale bar is missing.
Fig. 4A, E: Number of replicates, quantitative and statistical analysis has to be added. Fig. 5: Quantitative analysis is missing.
Fig. 6: Number of replicates, quantitative and statistical analysis has to be added.

© 2021. Published by The Company of Biologists under the terms of the Creative Commons Attribution License (https://creativecommons.org/licenses/by/4.0/).

Fig. 7G: EIPA is not mentioned either in the main text or in methods or in figure legend.

Fig. 7E, H: as suggested in the major comments, JIP4-overexpression in WT cells and in JIP-4KO cell has to be performed to confirm JIP4 activity on dextran recycling.

Significance

The authors identify in the adaptor protein JIP4, which physically interacts with the PI3P- binding protein Phafin2 on macropinosome, a new player in the general mechanism of recycling from macropinosomes. They identify the domain of JIP4 that specifically interacts with Phafin2.

The authors are fully aware of the literature regarding JIPs, Phafins and macropinosome recycling, that they appropriately cite, and apply different approaches to support their claims. Furthermore, they identify a phosphoinositide-driven mechanism, the PI3P-Phafin2-JIP4- dependent macropinosome tubulation, that has not been described previously for this trafficking pathway.

An audience interested in intracellular membrane trafficking and phosphoinositide functions would find this manuscript very interesting since it adds valuable information about a specific trafficking pathway.

My field of expertise is membrane trafficking and phosphoinositide metabolism.

Author response to reviewers' comments

General comments to the reviewers
We thank the reviewers for their thorough and helpful review of our manuscript. A detailed point-by-point response is included, and during the revision we will make the following changes that address comments from all reviewers:

- We will include more quantification and statistical analyses
- To strengthen the biological relevance, we will include experiments in additional cell lines. In particular, we will include HT1080 cells (which has been used in previous studies of JIP4) and perform siRNA-based depletion of JIP4.
- We will perform endogenous staining for JIP4 and tubule markers

Reviewer #1

Evidence, reproducibility and clarity

In this manuscript, the authors showed that JIP4 and Phafin2 are recruited to vesicular structures and interact with each. Knocking out of Phafin2 decreased the vesicular localization of JIp4. The authors further showed that JIP4 is recruited to tubular structures by Phafin2 and potentially regulates the recycling of macropinosomes. Overexpression of JIp4 promoted the tubulation of macropinosomes, while knocking out of JIP4 inhibited the endosome tubulation and retained the macropinosome cargoes in the endolysosomal pathway. Although there are a few experiments and quantification need to be improved to better support the conclusions, overall the data presented in this manuscript is convincing.

The major technical weakness of this manuscript is that most of the experiments in this study were based on observation made with over-expression of tagged plasmids. The overexpression system could profoundly affect the localization of the target protein. One example is with the GFP tagged JIp3 in figure 4, where the authors showed a diffused pattern and it's unclear whether it represents the normal localization of JIP3 or it was an overexpression artefact.

The majority of the presented JIP4 data was performed using lentivirus-generated stable cell lines expressing low amounts of JIP4 from the weak PGK promoter.

© 2021. Published by The Company of Biologists under the terms of the Creative Commons Attribution License (https://creativecommons.org/licenses/by/4.0/).

During the revision, we will generate a stable JIP3 line with a similar, weak expression level using lentiviral transduction.

The only staining of endogenous JIP4 is in figure 6 A, and the image presented did not support the tubule localization of JIP4. Moreover, all the observations were made in RPE-1 cell line. The authors should at least perform experiments to look into whether this interaction is conserved in different cell types.

We will perform additional experiments in other cell lines to strengthen our conclusions.

Specific points are listed as below

1. Figure 1 B-D, it is unclear how was the quantification performed. What is the replication number? Please include statistics analysis.

For Figure 1B and 1D (yeast 2-hybrid), we will add the calculation of relative reaction rates from absorbance values to the Materials and Methods, and modify the figures to show 3 independent experiments and the relevant statistical analysis. For Figure 1C, the relevant mass spec data are included in Suppl Table S2 of the main manuscript and we will reformat the document to show the origin of data plotted in Figure 1C.

2. Figure 1 F, please include input blots for JIP4. Please compare the pull down of Phafin2 with JIP4 and JIP3.

We will provide the input blot for the JIP4 pulldown in a supplemental figure in the revised manuscript. Based on our additional data points (2-hybrid assays, proteomics data, in-vivo data), we find no evidence that JIP3 is a potential interactor, and we do not believe that this negative datapoint will add to the manuscript. The Phafin2 binding site of JIP4 is poorly conserved in JIP3 (we will add an alignment for the relevant region formatted similarly to Figure 3b).

3. Figure 1 G and I do not support that Phafin2 recruit JIP4. It seems that Phafin2 and JIP4 are recruited simultaneously by other protein. Please discuss.

In previous work, we have extensively characterized the recruitment mechanism of Phafin2. Phafin2 is a direct lipid binder, and mutation of the lipid-binding domains of Phafin2 completely abolished membrane recruitment [1]. In order to strengthen the model that Phafin2 is required to recruit JIP4, we will test if JIP4 is recruited if these mutants instead of WT Phafin2 is expressed.

However, in light of our findings that Phafin2 knockout abrogates JIP4 recruitment (Figure 4C and 4D), whereas overexpression of Phafin2 leads to massive recruitment of JIP4, a direct recruitment is the most plausible explanation.

4. Figure 1 J only quantified 4 macropinosomes. Please include more macropinosomes for the quantification.

We will increase the sample size for this.

5. Please analyze and discuss what phospholipid (PI(3,4,5)P3 or PI(3,4)P2 or PI(4,5)P2) does the phafin2 PH domain and interact with. Please analyze and discuss whether JIP4 is recruited to PI(3,4,5)P3 or PI3P.

The Phafin2 lipid-binding activity is analyzed in detail in Schink et al, 2017 [1], as well as Tang et al. 2017 and 2020 [2, 3] and Matsuda-Lennikov et al. 2014 [4]. The Phafin2 PH domain binds to monophosphorylated phosphoinositides, PtdIns3P, PtdIns4P and PtdIns5P, and does not bind to any other phosphoinositide species. In addition, the Phafin2 FYVE domain binds only to PtdIns3P. JIP4 does not have a lipid-binding domain and has never been described in the literature to bind to any lipid.

6. Figure 2 B, it is unclear how did the authors performed the quantification. How did the authors determine the JIP4 signal "inside EEA1 positive vesicles"?

We used an ImageJ script to segment our acquired images using the EEA1 staining to generate regions of interest corresponding to EEA1 positive vesicles. We will clarify the Materials and Methods and publish the used code (ImageJ scripting code) on Github.

7. Figure 3 A, please label the plot to better indicate what are the x and y axis showing.

We will modify the figure.

© 2021. Published by The Company of Biologists under the terms of the Creative Commons Attribution License (https://creativecommons.org/licenses/by/4.0/).

8. Figure 3 b, please include sequence alignment of the JIP3/4.

We will modify the figure.

9. JIP3 seemed to be largely diffused even in WT RPE1 cells. This brings the question that whether the GFP-JIP3 overexpression altered the localization of JIP3.

As mentioned above, we will use lentiviral transduced stable cell lines expressing very low levels of JIP3 tagged with the same fluorophore as JIP4 to assess localization. However, we would like to highlight that Bonet-Ponce et al. 2020 - in agreement with our work - did not observe any vesicle localization of JIP3 [5]. Moreover, we have shown by yeast two-hybrid (Suppl. Table 1 and Figure 3C) that there is no indication of an interaction between JIP3 and neither Phafin1 nor Phafin2, and the identified Phafin2 binding motif is not conserved in JIP3. Also, in the presented LAPtag and APEX2 mass-spec data, we do not find JIP3 (Suppl Table S2 and S3).

10. Figure 4, the authors should provide localization evidence to show the JIP4 positive vesicles colocalize with dextran to prove that JIP4 is indeed recruited to macropinosomes.

We have already performed initial dextran uptake assays and can find that JIP4 is indeed localizing to macropinosomes containing a fluid-phase marker. We have attached this preliminary data (Temp Figure 3) and will perform additional quantifications.

The authors should also label clathrin dependent vesicles and show that JIP4 do not colocalize with clathrin dependent vesicles.

We have not made the claim that JIP4 does not localize at vesicles derived from Clathrin-dependent endocytosis, and we also note that macropinosomes and clathrin-derived endosomes follow the same maturation pathway after uncoating from actin or clathrin respectively.

11. The tubule localization of phafin2 and JIp4 presented in figure 4 C is not convincing. How did the authors distinguish between the tubules and a smaller vesicle adjacent to a large vesicle? Please clarify.

The quantifications reported in Figure 4C and 4D were derived from live-cell imaging movies. The imaging interval of 3sec (as reported in the materials and methods) is frequent enough that tubulation events can be easily distinguished from the approach of a second 2xFYVE positive vesicle. As an example, Figure 5B in the manuscript shows a timelapse montage of a tubulation event using the same marker and imaging interval. We have also attached more images (Temp Figure 1) using this marker, and the tubules are clearly different from small endosomes that are visible in the expanded images.
We will also provide more data on the tubule localization of JIP4.

12. Please co-express JIp4 and Phafin2 and show the relative tubule localization of both proteins.

This data is already shown in the present manuscript (Figure 4e), but we will include additional images and quantifications, as well as movies.

13. Figure6, please quantify the co-localization of JIP4 and the indicated markers of recycling endosomes.

We will show additional quantifications.

14. Figure 7, please provide high-content image for dextran uptake assay.

We will show additional images of the dextran uptake assays in the supplemental figures.

15. Figure 8, it is unclear whether the altered dextran "retention" is caused by altered recycling or degradation. The authors should perform experiments to probe these two possibilities.

We will design and perform experiments to investigate these two possibilities.

Minor

Please label Figure 2 D.

We will do so.

© 2021. Published by The Company of Biologists under the terms of the Creative Commons Attribution License (https://creativecommons.org/licenses/by/4.0/).

Reviewer #1, Significance

The role of JIP4 in endosome tubulation is well documented. It has been shown that ARF6 recruits JIP4 on macropinosomes and regulates the tubule formation. Although the authors discovered another mechanism by which JIP4 might be recruited to macropinosomes, they did not show any major novel functions of JIP4. (PMID 30969891, 26504170)

While the model of membrane recruitment of JIP4 by ARF6 has been proposed in the review literature, there is little to no primary data showing this. We would like to point out that neither of these two references shows or even implies that JIP4 is recruited to macropinosomes or endosomal tubules by ARF6. Marchesin et al. 2015 (PMID 26504170) – cited by this reviewer – explicitly proposes that ARF6 acts in trans from the plasma membrane (see Figure 7F and G of Marchesin et al., 2015) to act on RAB7 late endosomes, not macropinosomes [6]. Moreover, Williamson et al, 2019 (PMID 30969891), the second paper cited by this reviewer does not show any ARF6-dependent JIP4 recruitment either [7]. The only experiment involving JIP4 is a knockdown experiment in which dextran uptake was quantified by microscopy (see Figure 5C of Williamson et al, 2019) but no localization of JIP4 (or JIP3) is shown, nor is the connection to ARF6 clarified. In addition, their model does not propose a role for JIP4 on any tubulating structure, their model instead proposes that JIP3 acts at an earlier time point during macropinosome formation. During this time, we observed that JIP4 is not localized at macropinosomes (Figure 1G and 1H), nor is it required for completion of macropinocytosis (Figure 7F). Thus, the reviewer's assertion that the role, function and molecular mechanisms of JIP4 at macropinosomes (and tubules) is well documented, or that ARF6 has been demonstrated to act as a JIP4 recruiter, is not accurate.
In addition, we show now additional data that the ARF6 binding domain of JIP4 – as identified by crystallographic studies by Montagnac et al. 2009 [8] and Isabet et al. 2009 [9], is not required for tubule localization. We generated the V416A and I421A ARF6-binding mutations in JIP4 – also as identified and characterized by Isabet et al. (see Figure 5 of Isabet et al. 2009). The resulting proteins still localized to endosome/macropinosome tubules. In contrast, JIP4 lacking the Phafin2 binding domain identified in our study but retaining the ARF6-binding domain did not show any localization to tubules. We attach this new data (Temp Figure 1).
We also highlight that, as we have written in our Discussion, the overlap between JIP3 and JIP4 remains rather unclear. Many studies do not clearly distinguish between the two homologs [10], use them interchangeably [11], report that double perturbations are necessary to induce a phenotype [12], or that silencing of JIP3 and JIP4 induce similar additive [13] and non-additive effects [6]. Our data that the phosphoinositide-binding recruiter Phafin2 discriminates between JIP4 and JIP3 thus constitutes novel insight into JIP4 function.

This research would be interested to researchers focusing on endosomal trafficking pathways. One concern is that the authors used RPE-1 cells which has limited physiological relevance. To draw broader interests from general audience, the authors should prove that the proposed mechanism remain conserved in other cell types as well as primary cells.

We will perform key experiments in other cell types to broaden the biological relevance. To this end, we will use HT1080 cells, macrophages/dendritic cells and primary cells.

Reviewer #2

Evidence, reproducibility and clarity

Summary:
In this study, Wee Tan and colleagues report that a coiled-coil-containing protein JIP4 is targeted to macropinosomes by the PI3P-binding protein Phafin2 to promote tubulation and recycling from these organelles.

© 2021. Published by The Company of Biologists under the terms of the Creative Commons Attribution License (https://creativecommons.org/licenses/by/4.0/).

The evidence presented in this manuscript, particularly with respect to imaging, is of high quality. The immediate conclusions are supported by the data, which is convincing and appears reproducible. It must be noted that use of SEM in place of SD for biological error determination is not appreciated, as it underplays the actual variation between cells and samples, particularly in imaging studies. Western Blot analysis is not of the highest quality (and lacks quantification), but is well supported the imaging studies.

We will change the presentation of the statistics data (to 95 % CI), and will quantify the western blots.

Major points of discussion and criticism:

1. Line 85: 'This was interesting since JIP4 and it homologue JIP3 have been implicated in macropinocytosis (ref 17), although their function has remained largely unknown.' I was expecting that the authors would go on to determine the function of JIP4 in this process. However, given the limited mechanistic insights underpinning the manuscript, particularly considering the prior findings reported in refs #17 and #26, the present manuscript falls short in this regard.

In contrast to the current literature -and specifically Ref. 17 (Williamson et al, 2019) [7], our data show that JIP4 is not needed for successful macropinocytosis (Figure 7F). Moreover, we now also show new data that JIP4 knockdown in HT1080 cells – the same cell line used in Ref. 17, results in increased dextran retention (Temp Figure 2). This is in line with our JIP4 knockout phenotype reported in RPE1 cells. These data are in contradiction to the data and proposed explanatory mechanisms of Ref 17 with regard to JIP4 and macropinocytosis. Our data instead identify a PtdIns3P-dependent recruitment mechanism that targets JIP4 to fully internalized macropinosomes where it participates in the formation of recycling tubules. In addition to the membrane recruitment data in the manuscript, we show new data (Temp Figure 1) that clarifies that the interaction of JIP4 with ARF6 is not required for tubule localization. We note also that Ref 26 (Marchesin et al. 2015) [6] cited by the reviewer implicates JIP3 or JIP4 (it is not clear which) in the formation of tubules from late endosomes, not macropinosomes.

2. Line 99: 'and that the local membrane environment is not required'. While it is clear from the data provided that the local membrane environment doesn't contribute to the interaction in question, it most likely contributes to the function of the resulting complex.

We agree with this reviewer that the local membrane environment is likely needed for function; however, in this experiment we specifically wanted to exclude that JIP4 and Phafin2 are merely co-recruited by (unknown) tubule-localized factors and that they are directly interacting.

In my view investigating this membrane context using the techniques already described in the manuscript, such as proximity-based biotinylation, would open the doors to understanding the mechanism through which this protein pair achieves tubulation and recycling of plasma membrane components in this vesicular pathway.

A more detailed study of the factors regulating tubulation and recycling is desirable, but beyond the scope of this article. However, we provide and characterise a novel mechanism by which JIP4 is coupled to the membrane of a specific organelle, the macropinosome. As highlighted above, we find that ARF6 binding is not required for tubule localization, but that the Phafin2-binding domain of JIP4 is indispensable. We show this new data in the attached figures (Temp Figure 1).

3. Line 113: My understanding is that the authors define macropinosomes based on their size and appearance. Given the wide variety of vesicular structures populating the endolysosomal system, its seems that the use of unique/canonical markers of macropinosomes should be implemented at least in some key experiments, such as those in Figure 1.

The canonical definition of a macropinosome is a vesicle between 0.2 and 10 μm in diameter (see Swanson et al, 2008), and there are no canonical markers that would be specific for macropinosomes and not endosomes. Macropinosomes mature and gain markers like early endosomes. Rabankyrin5 has been described as marker for macropinosomes, but is also found on other vesicles. Nevertheless, we now show JIP4 tubules forming on vesicles

© 2021. Published by The Company of Biologists under the terms of the Creative Commons Attribution License (https://creativecommons.org/licenses/by/4.0/).

containing 70kDa dextran, which is a specific marker for macropinocytic uptake (Temp Figure 3).

4. Line 150: 'This suggests that their interaction is specific.' The authors present compelling evidence that JIP4 and Phafin2 make a unique pairing with respect to their homologues. However, considering that the interaction between these proteins forms the central focus of the manuscript, it is suggested that the authors take this opportunity to more closely define the nature of this interaction based on the sequence similarities/difference amongst the homologues.

In order to map the interaction with JIP4 and Phafin2 in more detail, we will generate chimeric proteins with their homologs JIP3 and Phafin1 and test if these are recruited to tubules. We have already performed experiments where we map the binding site of JIP4 and Phafin2, and deletion of this binding site abrogates tubule localization (Temp Figure 1).

5. Line 217, Fig. 7G-I: The authors' findings regarding increased intracellular Dextran levels do not correspond with data presented in ref #17 in what I understand to be largely the same experiment. Is this a cell-type specific effect, or is there an alternate explanation?

We are aware of this discrepancy and will increase the generalizability of our conclusions with additional experiments and additional cell lines. We have already performed knockdown experiments, using two independent siRNAs, in HT1080– the cell line used in ref. #17 – and find, in line with our RPE1 knockout data – increased dextran retention. We have attached this data (Temp Figure 2).

6. No working model is presented to summarise the key findings of the study.

We will present a model in the revised manuscript.

Minor points:

D is missing in Figure 2

We will correct this.

Some form of quantification should be provided in figure 3E and F

We will perform additional quantifications.

Supplementary Fig. 1 has display panels A-D, which are not matched by the legend.

We will correct this.

Reviewer #2, Significance

The manuscript is rather observational in nature. The scope of the study is rather limited, considering that it connects 2 proteins already known to function within the endocytic pathway. In its present form, the study neither identifies new endocytic players nor elaborates on the surrounding molecular context pertaining to the collaboration between JIP4 and Phafin2 in macropinocytosis. Taking this together with prior literature on JIP4 in macropinocytosis and endocytic recycling (references #17 and 26), the present findings do not present substantial conceptual or mechanistic advances in my view.

I would recommend that the authors pursue new mechanistic insights, as noted under Major point #2, and evaluate consequences of JIP4 inactivity on the abundance/function of cell surface proteins subject to the macropinocytosis pathway to bolster the impact of their study.

We agree with the reviewer that further details on the molecular context would be valuable. In our newly attached data, we provide data on JIP4 mutants that are aimed at dissecting the molecular players involved (Temp Figure 1). We will test these JIP4 mutants and additional mutants that we will generate, in functional experiments. This will clarify the molecular partners required for specific functions of JIP4. The proposed experiments (BioID / APEX of endosomal tubules) are attractive, but clearly beyond the scope of this manuscript as it is a rather large project on its own.

However, we also note that the present findings stand in contradiction to the prior literature on the function of JIP4 in macropinocytosis (Ref 17, Williamson et al, 2019). The specific claim of Ref 17 is that JIP3 is recruited by ARF6 on the plasma membrane (localization not shown) to complete the formation of macropinosomes [7]. The involvement of JIP4 in macropinocytosis was assumed to be similar to JIP3 based on their common ability to bind ARF6 and that knockdown of either JIP3 or JIP4 reduced dextran

© 2021. Published by The Company of Biologists under the terms of the Creative Commons Attribution License (https://creativecommons.org/licenses/by/4.0/).

uptake. This linkage of JIP4 and macropinocytosis in Ref 17 is extremely tenuous and supported by little evidence. We have directly quantified the success rate of macropinosomes and show that JIP4 ablation does not impair macropinocytosis. We have also performed dextran assays and we do not find the same effect as Ref 17, using both knockout and siRNA disruption of JIP4. Instead, the collective data reported in this manuscript implicate JIP4 in recycling from fully internalized macropinosomes, and provide partial insight into the molecular mechanisms.

Ref. 26 (Marchesin et al. 2015) does provide evidence for a role of JIP3 and/or JIP4 (not distinguished) in late endosome tubulation in close proximity to the plasma membrane [6]. However, the tubulation mechanism proposed where ARF6 acts in trans from the plasma membrane to modulate JIP3 or JIP4 on the endosome membrane is inapplicable to macropinosomes, which are frequently >1µm in diameter. This is readily visible in our CLEM image and model (Figure 5C, D), where the tubule is several sections (hundreds of nm) away from plasma membrane. Additionally, in the model proposed by Ref 26, the mechanism by which JIP3 or JIP4 would exert force on (or even be associated with) the endosome membrane is entirely absent. We restrict our claim in this manuscript, that Phafin2 is the membrane anchor for JIP4 but not JIP3, specifically to macropinosomes (for which we present data).

Furthermore, we note also that in other work involving JIP3 or JIP4 and endocytic recycling or recycling compartments, the membrane anchor is not identified and the role of ARF6 is described as a modulatory partner [8, 11]. The functional overlap of JIP3 and JIP4 also remains unclear [6, 7, 11-13]. The present findings that Phafin2 is a phosphoinositide-dependent membrane anchor that is specific for the JIP4 isoform may therefore be of interest to a trafficking audience.

Reviewer #3

Evidence, reproducibility and clarity

Summary:

In the manuscript entitled "JIP4 is recruited by the phosphoinositide-binding protein Phafin2 to promote recycling tubules on macropinosome", Kia Wee Tan and colleagues show that the adaptor protein JIP4 physically interacts with the PI3P-binding protein Phafin2. The authors indicated the sites of interaction between Phafin2 and JIP4 as sites on macropinosomes where recycling tubules originate. This interaction is required for JIP4 localization on macropinosomes and is specific for JIP4, since its homologue JIP3 does not bind to Phafin2 and is not recruited to macropinosomes. In addition, they show that PI3P is required for the localization of Phafin2 and hence of JIP4 on macropinosomes. The authors also generated a JIP4-KO cell line that they used with live and fixed samples to perform functional studies on JIP4 that led the authors to the conclusion that JIP4 is involved in recycling from macropinosomes, since in the absence of JIP4 the cargo they used to track macropinosomes (10KD dextran) is retained within intracellular organelles.

Major comments:
The authors build their manuscript on JIP4 on their previous observation that Phafin2 is a novel regulator of macropinosome formation (ref 16.) However, this observation is only available at bioRxiv as an unreviewed manuscript since 2017 (Schink, K.O., et al., The PtdIns3P-binding protein Phafin2 escorts macropinosomes through the cortical actin cytoskeleton. 2017, bioRxiv) Is there a more updated or different and reviewed version of these data?

This manuscript is currently still under review. The parts relating to the JIP4-Phafin2 interaction and the Phafin2 recruitment mechanism are not affected by this revision and are still consistent with the version published on BioRxiv. A revised version will be provided upon resubmission of this manuscript for reference.

While the interaction of JIP4 and Phafin2 is nicely studied and reported, the major problem I see concerns the conclusion that JIP4 localizes on tubules and promotes tubulation and recycling from macropinosomes. In fact, tubules are not clearly visible in any of the images reported in the Figures. These are visible only for some of the JIP4-positive structures in suppl. Video1. Instead, the figure images clearly show that the colocalization of JIP4 with macropinosomes is restricted to

© 2021. Published by The Company of Biologists under the terms of the Creative Commons Attribution License (https://creativecommons.org/licenses/by/4.0/).

specific domains. In many instances it is difficult to discriminate whether these domains pertain to the macropinosome surface or are instead endosomes tethered to/fusing with macropinosomes. The authors should provide much more convincing evidence of JIP4 association with/effect on tubulation at macropinosomes.

We agree with the reviewer that our main figures do indeed show data characterizing JIP4 positive tubules without first providing evidence that these JIP4 structures that we describe are tubules. This is an oversight in the preparation of the manuscript and we will furnish more extensive fluorescence microscopy evidence of JIP4 on macropinosome tubules. We have also attached some data (Temp Figure 1), where the tubule localization is shown much more clearly.

In the main manuscript, we have performed correlative light-electron microscopy and show that JIP4-positive tubules are emerging from vesicles in Figure 5. Moreover, JIP4 colocalizes with tubule markers (2xFYVE$^{(WDFY2)}$, Retromer). However, we will provide additional CLEM images from independent preparations and perform super-resolution imaging to visualize the tubule association of JIP4 more clearly. Our data also shows that JIP4 enhances tubulation of macropinosomes (Figure 7).

The functional studies on JIP4-KO cells do not result in any conclusive evidence since a block of macropinosomal recycling should lead to an increase in macropinosome area, which is not reported among the phenotypes induced by JIP4-KO. The authors instead describe that the cargo they use to trace macropinocytosis, i.e. 10KD dextran, accumulates in JIP4-KO cells 30 min after internalization. Here there are three points the authors should/could consider:

1. The accumulation of dextran occurs in compartments that do not appear, judging by the images, to be macropinosomes but rather endosomal structures. Higher resolution images corroborated by morphometric analysis are necessary

We will provide high resolution images and additional measurements. In the majority of experiments, we can directly track macropinosomes from the moment of their formation. However, we will provide additional data, measurements, and movies.

2. 70KD dextran (internalized exclusively by macropinocytosis) would be preferable to 10KD dextran since the latter can be internalized also by fluid phase and clathrin-mediated endocytosis. The use of EIPA to discriminate macropinocytosis from endocytosis is questionable since EIPA inhibits other forms of internalization under certain conditions (Canton J, Front Immunol 9: 2286) .

We share the concerns of this reviewer regarding the effects of EIPA, but -maybe unfortunately - it is still considered one of the “standard” assays to evaluate macropinocytosis. To corroborate our findings, we will test newly-reported macropinosome inhibitors (Lin et al 2019) to bolster our data. We have used 70kDA dextran in live-cell imaging and we find that JIP4 labels dextran-positive compartments (Temp Figure 3). Moreover, new flow cytometry data used 70 kDA dextran and found the same phenotype after siRNA mediated knockdown of JIP4 (Temp Figure 2).

In addition the authors do not show the impact of JIP4 depletion on macropinocytosis (as the number of macropinosomes/cell), and they do not clearly demonstrate that the higher amount of intracellular dextran is due to defective recycling rather than increased internalization.

We will use live cell imaging and automated tracking to score the number of macropinosomes formed in control and knockout cells. This will complement our current data, where we show that JIP4 KO does not impair the success rate of macropinosome formation. Moreover, given that JIP4 does not localize to forming macropinosomes (Figure 1G, H) but only arrives after establishment of endosomal identity, it is unlikely that JIP4 should affect macropinosome formation.

Furthermore, given the expertise of the authors with live imaging, the visualization of JIP4-positive tubules carrying the cargo (dextran) would add strong evidence that the JIP4-positive tubules are actually used for the recycling of cargos. For example, dextran recycling and release by WT and JIP4-KO cells could also be measured by TIRF microscopy.

We will perform live cell imaging of dextran-labelled cells to visualize JIP4-positive tubules and dextran. In addition, we will test and image other cargoes.

In addition, the functional role of JIP4 in cargo recycling from early macropinosomes has been tested in WT and JIP4-KO cell lines. In this case it would be beneficial for the claim and for the

© 2021. Published by The Company of Biologists under the terms of the Creative Commons Attribution License (https://creativecommons.org/licenses/by/4.0/).

specificity of JIP4-mediated cargo recycling to perform a rescue experiment in JIP4-KO cells by overexpressing JIP4.

We will perform live cell imaging of dextran uptake and recycling. Moreover, we will perform a rescue experiment.

In addition, we are in the process of performing functional experiments using siRNA-based JIP4 depletion. We have already concluded one set of experiments in HT1080 cells, which show the same phenotype as we report for the knockout cell lines (Temp Figure 2).

3. They should at the same time follow an endocytic tracer to exclude an impairment in endocytic (and not specific macropinocytic) recycling.

We do not propose that the observed defects are specific for macropinosomes. It is rather likely that other vesicles at the same maturation stage show JIP4-dependent recycling. However, we will also broaden our experiment to include other endocytic cargoes.

Although the authors show by different means the physical and functional interaction of Phafin2 and JIP4, most of the data lack quantitative analysis and statistical evaluation of the results (see Fig.3E, F; 4A, B; Fig.5 and Fig.6 and see minor comments).

In most cases they show colocalization of JIP4 with other markers by plotting the fluorescence intensities of the two proteins in a Region of Interest without providing any quantitative or statistical analysis, the results shown in Fig.3E, F; 4A, B; Fig.5 and Fig.6 cannot be more than observations.

We will perform the requested quantitative analysis. For the presented CLEM data, the sample size is low due to the laborious sample preparation, however, we will present additional data from independent experiments and EM sample preparations.

A general consideration regards the use of a single cell line (RPE1) and a single JIP4-KO line (RPE-JIP4-KO) that raises concerns about how general (or cell-specific) are the claims proposed in the manuscript. Testing the model described in the manuscript in a different cell line and, at least, in two other JIP4-KO clones (or in RPE1 treated with siRNAs against JIP4) would add substantial strength to the manuscript. The suggested supplemental experiments are fully in line with the expertise of the authors and with the methods already applied in the manuscript and can be completed in 3-6 months.

We have already performed knockdown experiments in HT1080 cells (shown as Temp Figure 2) and will perform additional experiments in other cell types.

Minor comments:

Fig. 1B-C-D: error bars and statistical analysis should be added.

We will correct this.

Fig. 1E: Number of replicates and statistical analysis has to be added.

We will correct this.

Fig. 1F: the authors should include the WB with anti-GFP or anti-JIP4 to show immunoprecipitation efficiency of the bait.

We will include anti-JIP4 in the supplemental figures.

Fig. 2C: The use of Rapamycin at 250nM for 30 minutes can reduce mTOR activity and thus induce autophagy. To exclude any interference of the mTOR/autophagic pathway, the authors should test Rapamycin at lower concentration/shorter time of treatment or include in the manuscript (as supplemental data) the evaluation of mTOR activity by measuring phosphorylation of S6-Kinase in RPE either untreated and treated with 250nM Rapamycin.

This experiment is done in the presence of the VPS34 inhibitor SAR405 (to displace Phafin2 from macropinosomes), which also blocks autophagy.

Fig. 3C-D: error bars and statistical analysis should be added.

We will include this.

Fig. 3E-F: Number of replicates, quantitative and statistical analysis has to be added.

We will include this.

© 2021. Published by The Company of Biologists under the terms of the Creative Commons Attribution License (https://creativecommons.org/licenses/by/4.0/).

Fig. 3F: scale bar is missing.
We will correct this.

Fig. 4A, E: Number of replicates, quantitative and statistical analysis has to be added.
We will include this.

Fig. 5: Quantitative analysis is missing.
The point of the CLEM was to show that the tubule is continuous with the limiting membrane of the macropinosome. We will add additional data in Supplemental figures.

Fig. 6: Number of replicates, quantitative and statistical analysis has to be added.
We will include this.

Fig. 7G: EIPA is not mentioned either in the main text or in methods or in figure legend.
We will correct this

Fig. 7E, H: as suggested in the major comments, JIP4-overexpression in WT cells and in JIP-4KO cell has to be performed to confirm JIP4 activity on dextran recycling.
We will include additional cell lines and a rescue experiment

Reviewer #3, Significance

The authors identify in the adaptor protein JIP4, which physically interacts with the PI3P-binding protein Phafin2 on macropinosome, a new player in the general mechanism of recycling from macropinosomes. They identify the domain of JIP4 that specifically interacts with Phafin2.

The authors are fully aware of the literature regarding JIPs, Phafins and macropinosome recycling, that they appropriately cite, and apply different approaches to support their claims. Furthermore, they identify a phosphoinositide-driven mechanism, the PI3P-Phafin2-JIP4-dependent macropinosome tubulation, that has not been described previously for this trafficking pathway.

An audience interested in intracellular membrane trafficking and phosphoinositide functions would find this manuscript very interesting since it adds valuable information about a specific trafficking pathway.

My field of expertise is membrane trafficking and phosphoinositide metabolism.
We thank the reviewer for his/her concise summary of the importance of our work, and for finding our manuscript very interesting for a membrane trafficking / phosphoinositide readership.

REFERENCES FOR REVIEWERS

1. Schink, K.O., et al., *The PtdIns3P-binding protein Phafin2 escorts macropinosomes through the cortical actin cytoskeleton*. bioRxiv, 2017: p. 180760.
2. Tang, T.-X., et al., *Structural, thermodynamic, and phosphatidylinositol 3-phosphate binding properties of Phafin2*. Protein Science, 2017. **26**(4): p. 814-823.
3. Tang, T.X., C.V. Finkielstein, and D.G.S. Capelluto, *The C-terminal acidic motif of Phafin2 inhibits PH domain binding to phosphatidylinositol 3-phosphate*. Biochim Biophys Acta Biomembr, 2020. **1862**(6): p. 183230.
4. Matsuda-Lennikov, M., et al., *Lysosomal interaction of Akt with Phafin2: a critical step in the induction of autophagy*. PLoS One, 2014. **9**(1): p. e79795.
5. Bonet-Ponce, L., et al., *LRRK2 mediates tubulation and vesicle sorting from lysosomes*. Sci Adv, 2020. **6**(46).
6. Marchesin, V., et al., *ARF6-JIP3/4 regulate endosomal tubules for MT1-MMP exocytosis in cancer invasion*. 2015. **211**(2): p. 339-358.
7. Williamson, C.D. and J.G. Donaldson, *Arf6, JIP3, and dynein shape and mediate macropinocytosis*. Molecular Biology of the Cell, 2019. **30**(12): p. 1477-1489.
8. Montagnac, G., et al., *ARF6 Interacts with JIP4 to Control a Motor Switch Mechanism Regulating Endosome Traffic in Cytokinesis*. Current Biology, 2009. **19**(3): p. 184-195.

© 2021. Published by The Company of Biologists under the terms of the Creative Commons Attribution License (https://creativecommons.org/licenses/by/4.0/).

9. Isabet, T., et al., *The structural basis of Arf effector specificity: the crystal structure of ARF6 in a complex with JIP4.* The EMBO Journal, 2009. **28**(18): p. 2835-2845.
10. Vilela, F., et al., *Structural characterization of the RH1-LZI tandem of JIP3/4 highlights RH1 domains as a cytoskeletal motor-binding motif.* Scientific Reports, 2019. **9**(1).
11. Montagnac, G., et al., *Decoupling of Activation and Effector Binding Underlies ARF6 Priming of Fast Endocytic Recycling.* Current Biology, 2011. **21**(7): p. 574-579.
12. Sato, T., et al., *JSAP1/JIP3 and JLP regulate kinesin-1-dependent axonal transport to prevent neuronal degeneration.* Cell Death & Differentiation, 2015. **22**(8): p. 1260-1274.
13. Gowrishankar, S., et al., *Overlapping roles of JIP3 and JIP4 in promoting axonal transport of lysosomes in human iPSC-derived neurons.* bioRxiv, 2020: p. 2020.06.13.149443.

First decision letter

MS ID#: JOCES/2021/258495

MS TITLE: JIP4 is recruited by the phosphoinositide-binding protein Phafin2 to promote recycling tubules on macropinosomes

AUTHORS: Kia Wee Tan, Viola Nahse, Coen Campsteijn, Andreas Brech, Kay Oliver Schink, and Harald Stenmark
ARTICLE TYPE: Research Article

We have now reached a decision on the above manuscript.

There are no additional reviewers reports as we have used those available from Review Commons to reach this decision. To see copy of this decision letter, please go to: https://submit-jcs.biologists.org and click on the 'Manuscripts with Decisions' queue in the Author Area. (Corresponding author only has access to reviews.)

As you know, the reviewers raise a number of substantial criticisms that would prevent me from accepting the paper at this stage but you have suggested a series of revisions that I consider would likely prove sufficient to address those concerns. We may then return it to the reviewers but will make that decision once we see the revision.

Specifically, including further validation in another cell line is welcome, as is use fo RNAi, publishing you analysis code and improvements to the figures. Overall, I do consider the level of new insight sufficient for publication in JCS and thank you for your clear arguments in that regard. Ensuring clarity with regard to your other work on bioRxiv (in particular any revised version) is also important. The new temporary figures are very useful and I encourage you to work these data into the revision as you see fit. Please do contact us should you require any further clarification on these points or any other issues during revision.

We are aware that you may be experiencing disruption to the normal running of your lab that makes experimental revisions challenging. If it would be helpful, we encourage you to contact us to discuss your revision in greater detail. Please send us a point-by-point response indicating where you are able to address concerns raised (either experimentally or by changes to the text) and where you will not be able to do so within the normal timeframe of a revision. We will then provide further guidance. Please also note that we are happy to extend revision timeframes as necessary.

Please ensure that you clearly highlight all changes made in the revised manuscript. Please avoid using 'Tracked changes' in Word files as these are lost in PDF conversion.

I should be grateful if you would also provide a point-by-point response detailing how you have dealt with the points raised by the reviewers in the 'Response to Reviewers' box. Please attend to

© 2021. Published by The Company of Biologists under the terms of the Creative Commons Attribution License (https://creativecommons.org/licenses/by/4.0/).

all of the reviewers' comments. If you do not agree with any of their criticisms or suggestions please explain clearly why this is so.

First revision

Author response to reviewers' comments

Comments to editor

We thank the reviewers and the editor for very insightful and helpful comments. A point-by-point response to all reviewer comments has been included below. Quantitative analysis requested by all three reviewers have been performed and reported alongside previous data. As highlighted in the invitation for revision, we have validated results in another cell line, used siRNA to supplement our genetic knockout, and provided our analysis code via Github. New data presented in the temporary figures have been presented as new figures in the revised manuscript. We have also clarified the relationship to our other manuscript submitted to bioRxiv, which does not overlap with the present manuscript. The other manuscript on bioRxiv contains some data from a two-hybrid screen. These will be removed from the revised version of the bioRxiv manuscript and are therefore included in the present JCS manuscript instead.

Please note that, while the guidelines for resubmission ask for all changes made in the manuscript to be highlighted, we have not done so, as the text changes to most sections are extensive. Because we have revised the figure layout and supplementary material naming, virtually all paragraphs contain at least minor alterations. In addition, the manuscript and figure layout has been reorganized for greater clarity and to accommodate the changes requested by reviewers.

Reviewer #1

In this manuscript, the authors showed that JIP4 and Phafin2 are recruited to vesicular structures and interact with each. Knocking out of Phafin2 decreased the vesicular localization of JIp4. The authors further showed that JIp4 is recruited to tubular structures by Phafin2 and potentially regulates the recycling of macropinosomes. Overexpression of JIp4 promoted the tubulation of macropinosomes, whilte knocking out of Jip4 inhibited the endosome tubulation and retained the macropinosome cargoes in the endolysosomal pathway. Although there are a few experiments and quantification need to be improved to better support the conclusions, overall the data presented in this manuscript is convincing.

The major technical weakness of this manuscript is that most of the experiments in this study were based on observation made with over-expression of tagged plasmids. The overexpression system could profoundly affect the localization of the target protein. One example is with the GFP tagged JIp3 in figure 4, where the authors showed a diffused pattern and it's unclear whether it represents the normal localization of JIp3 or it was an overexpression artefact. The only staining of endogenous JIP4 is in figure 6 A, and the image presented did not support the tubule localization of JIP4. Moreover, all the observations were made in RPE-1 cell line. The authors should at least perform experiments to look into whether this interaction is conserved in different cell types.

We have previously reported endogenous staining of JIP4 in Figure 2A (also in the revised manuscript), and include new data on endogenous staining of JIP4 in Figure 2E and S2 of the revised manuscript. New data using HT1080 cells in functional assays is included in Figure 4. We address JIP3 in our reply to Comment #9.

1. Figure 1 B-D, it is unclear how was the quantification performed. What is the replication number? Please include statistics analysis.

For Figure 1B and 1C, 3 experiments were performed and datapoints now shown. Figure S1A (formerly Figure 1C) is a semi-quantitative mass spectrometry experiment and was performed once. All individual replicate datapoints, means and S.E.M. have been added to Figure 1B and 1C, however the results of these are fairly binary (interact/does not interact).

© 2021. Published by The Company of Biologists under the terms of the Creative Commons Attribution License (https://creativecommons.org/licenses/by/4.0/).

2 .Figure 1 F, please include input blots for JIP4. Please compare the pull down of Phafin2 with JIP4 and JIP3.
Input blot has been added in Figure 1D. Note that the GFP control has a larger molecular weight than GFP alone (which is 27kDa) because it also contains the purification tag.

3. Figure 1 G and I do not support that Phafin2 recruit JIP4. It seems that Phafin2 and JIP4 re recruited simultaneously by other protein. Please discuss.
The recruitment of Phafin2 to membranes has been analyzed in detail in our previous work (Schink et al., 2017), as well as work from other labs (Matsuda-Lennikov et al., 2014; T.-X. Tang et al., 2017; T. X. Tang, Finkielstein, & Capelluto, 2020), and is dependent on lipid binding. Our Phafin2 knockout data further shows that JIP4 recruitment is dependent on Phafin2. We have reworded this part to report the dynamic colocalization only and placed our interpretations further below.

4. Figure 1 J only quantified 4 macropinosomes. Please include more macropinosomes for the quantification.
We have now quantified 17 macropinosomes and report this data in Figure 1I.

5. Please analyze and discuss what phospholipid (PI(3,4,5)P3 or PI(3,4)P2 or PI(4,5)P2) does the phafin2 PH domain and interact with. Please analyze and discuss whether JIP4 is recruited to PI(3,4,5)P3 or PI3P.
The PH domain of Phafin2 binds to PtdIns3P, PtdIns4P, and PtdIns5P (Matsuda-Lennikov et al., 2014; T.-X. Tang et al., 2017; T. X. Tang et al., 2020). JIP4 is not reported to bind to any phosphoinositide, and we do not make this claim in the paper.

6. Figure 2 B, it is unclear how did the authors performed the quantification. How did the authors determine the JIP4 signal "inside EEA1 positive vesicles"?
We used an ImageJ script that segments and generates Regions of Interest using the EEA1 channel. The script has been made available on Github and this is now indicated in Materials and Methods.

7. Figure 3 A, please label the plot to better indicate what are the x and y axis showing.
8. Figure 3 b, please include sequence alignment of the JIP3/4.
We have replaced Figure 3A to show sequence alignment of the JIP4 Phafin2 binding region with JIP3. Figure 3B is unchanged except for minor formatting. The full alignments have been added as Figure S3A and S3B.

9. Jip3 seemed to be largely diffused even in WT RPE1 cells. This brings the question that whether the GFP-JIp3 overexpression altered the localization of Jip3.
We have also cloned mNG-JIP3 to compare with JIP4 using the same fluorophore. The localization is unchanged. In Figure 5E, the localization of JIP4 is very different from JIP3, despite identical acquisition settings and roughly similar cytoplasmic concentrations. We have also generated chimeras of JIP3 and JIP4 and, using these chimeras, pinpoint the regions required for recruitment of JIP4 to tubules. The chimera experiment reported in Figure 5E and 5F provides evidence that the affinity difference between JIP4 and JIP3 is massive. In line with this, we note that another lab studying LYTL (Lysosomal Tubulation driven by LRRK2) also shows a cytosolic localization of JIP3 in their supplementary data (Bonet-Ponce et al., 2020).

10. Figure 4, the authors should provide localization evidence to show the JIP4 positive vesicles colocalize with dextran to prove that JIp4 is indeed recruited to macropinosomes. The authors should also label clathrin dependent vesicles and show that JIP4 do not colocalize with clathrin dependent vesicles.
We now show JIP4 on vesicles labeled with 70kDa Dextran in Figure 1E. We have not made any claims about JIP4 on clathrin-dependent vesicles. Moreover, since both macropinosomes and clathrin-dependent vesicles mature to endosomes, it is probable that these vesicles behave similarly.

11. The tubule localization of phafin2 and JIp4 presented in figure 4 C is not convincing. How did the authors distinguish between the tubules and a smaller vesicle adjacent to a large vesicle? Please clarify.

© 2021. Published by The Company of Biologists under the terms of the Creative Commons Attribution License (https://creativecommons.org/licenses/by/4.0/).

In the referenced Figure 4C, which is now Figure 5A in the revised manuscript, these tubules were observed emerging from the limiting membrane by timelapse microscopy, similar to the montage shown in Figure 6B. The time interval between frames in all videos taken is 3 seconds, which is sufficiently fast to distinguish between approaching vesicles and emerging tubules.

12. Please co-express JIp4 and Phafin2 and show the relative tubule localization of both proteins.
A co-expression is reported in Figure 1F, 1H, Movie 1, and in Figure 6E and 6F (formerly Figure 7A and 7B). Figure 6F highlights a tubule emerging from a bright Phafin2 subdomain. This is quantified in the intensity lineplot of Figure 6G.

13. Figure 6, please quantify the co-localization of JIp4 and the indicated markers of recycling endosomes.
These data have now been quantified and reported in Figure 7. We have replaced the SIM panel (formerly Figure 6A) with regular microscopy images.

14. Figure 7, please provide high-content image for dextran uptake assay.
It is not clear to us what “high-content” image means in this context. The images displayed are indeed cropped from the original field of view, however this is only to remove unnecessary black space for display. We can provide uncropped images, but they do not contain any more information than what is already shown, as the field of view usually only fits one cell fully. We did not acquire low magnification images.

15. Figure 8, it is unclear whether the altered dextran "retention" is caused by altered recycling or degradation. The authors should perform experiments to probe these two possibilities.
We agree that this is an important point and now show new data that macropinocytic internalization is not altered by JIP4 ablation. We also show new data that dextran is retained using siRNA depletion, in Figure 4 of the revised manuscript. We have laid out our interpretations of the data and alternative possibilities in the discussion.

Minor

Please label Figure 2 D.

Label included in the revised manuscript.

Reviewer #2

In this study, Wee Tan and colleagues report that a coiled-coil-containing protein JIP4 is targeted to macropinosomes by the PI3P-binding protein Phafin2 to promote tubulation and recycling from these organelles.

The evidence presented in this manuscript, particularly with respect to imaging, is of high quality. The immediate conclusions are supported by the data, which is convincing and appears reproducible. It must be noted that use of SEM in place of SD for biological error determination is not appreciated, as it underplays the actual variation between cells and samples, particularly in imaging studies. Western Blot analysis is not of the highest quality (and lacks quantification), but is well supported the imaging studies.
We have replaced SEM with 95% confidence interval in our graphs and plots. The exceptions are in the yeast two-hybrid graphs where the information is fairly binary (interacts/does not interact).

1. Line 85: 'This was interesting since JIP4 and it homologue JIP3 have been implicated in macropinocytosis (ref 17), although their function has remained largely unknown.' I was expecting that the authors would go on to determine the function of JIP4 in this process. However, given the limited mechanistic insights underpinning the manuscript, particularly considering the prior findings reported in refs #17 and #26, the present manuscript falls short in this regard.
In contrast to the current literature and specifically Ref. 17 (Williamson & Donaldson, 2019), our data show that JIP4 is not needed for successful macropinocytosis (Figure 4B, formerly 7F). In the revised manuscript, we add new data as Figure 4C and 4D showing that ablation of JIP4 does not change the rate or size of macropinosome formation. These data are not in agreement with the

© 2021. Published by The Company of Biologists under the terms of the Creative Commons Attribution License (https://creativecommons.org/licenses/by/4.0/).

model proposed in Ref 17 and place the function of JIP4 in macropinocytosis after the internalization process is complete.
We now also show new data that JIP4 knockdown in HT1080 cells – the same cell line used in Ref. 17, results in increased dextran retention (Figure 4G). This is in line with our JIP4 knockout and knockdown phenotypes reported in RPE1 cells (Figure 4E and 4F). Our data instead identify a PtdIns3P- and Phafin2-dependent recruitment mechanism that targets JIP4 to fully internalized macropinosomes where it participates in the formation of recycling tubules. This is tested with several converging lines of evidence in vitro and in cells.
With regard to Ref 26 raised by the reviewer, we note that Marchesin et. al., 2015 firstly implicate JIP3 or JIP4 in the formation of tubules from a different organelle (the late endosome), and secondly, propose a mechanism that cannot operate as described from the macropinosome (Marchesin et al., 2015). The model proposed by Marchesin and co-workers (Figure 7F in Marchesin et al) suggests that Arf6 acts in trans from the plasma membrane, and the recruitment mechanism of JIP3/JIP4 to endosomal tubules is not explored. In contrast, we investigate how JIP4 is recruited to tubulating vesicles. Moreover, the JIP4 tubules we show are located far from the plasma membrane (Figure 6D shows a thin slice from a much rounder macropinosome). Therefore, either we report on a different phenomenon than Ref 26, or the model proposed in Ref 26 is in need of refinement.
The revised manuscript also adds new data that JIP4 does not require ARF6 to target macropinosome tubules (Figure 5C and 5D), and that Phafin2-dependent tubule targeting is not conserved in JIP3 (Figure 5E and 5F). The implications of these datapoints are explored further in our discussion. Taken together, the manuscript thus contributes both positive and negative mechanistic insight to the function of JIP4.

2. Line 99: 'and that the local membrane environment is not required'. While it is clear from the data provided that the local membrane environment doesn't contribute to the interaction in question, it most likely contributes to the function of the resulting complex. In my view investigating this membrane context using the techniques already described in the manuscript, such as proximity-based biotinylation, would open the doors to understanding the mechanism through which this protein pair achieves tubulation and recycling of plasma membrane components in this vesicular pathway.
We have added new data in Figure 5C-F. These data support that targeting of JIP4 to macropinosome tubulation zones is Phafin2-dependent, ARF6-independent, and not conserved in the JIP3 homolog. The experiments proposed are interesting but beyond the scope of this manuscript.

3. Line 113: My understanding is that the authors define macropinosomes based on their size and appearance. Given the wide variety of vesicular structures populating the endolysosomal system, its seems that the use of unique/canonical markers of macropinosomes should be implemented at least in some key experiments, such as those in Figure 1.
Macropinosomes were originally defined as “large (diameter greater than 0.2 mm) heterogeneous phase-bright organelle observed to emanate from the base of waving sheet-like extensions of the plasma membrane called ruffles.” (Kerr & Teasdale, 2009). The same authors state further that “In the absence of any specific markers or cargoes, this definition remains perhaps our most accurate descriptor of this enigmatic process.”.
The structures that we report in live imaging data come from large vesicles entering from ruffling regions of the plasma membrane. These vesicles can be observed in Movie 1-3. The size of these vesicles (>500nm) and their formation are visibly distinct from other routes of internalization such as clathrin, caveolin, and endophilin-dependent pathways, which are <200nm. Those vesicles would be below the resolution limit and would therefore not have visible lumens. There are currently no markers that are known to be unique for macropinosomes. We have limited our interpretation to macropinosomes as our data are based on these structures and we do not also claim that JIP4 is exclusive to macropinocytosis. In order to further distinguish the structures we observed from other vesicle classes, we show in Figure 1E in the revised manuscript that JIP4 accumulated on large vesicles that contain 70kDa dextran, which is selective for macropinocytic internalization.

4. Line 150: 'This suggests that their interaction is specific.' The authors present compelling evidence that JIP4 and Phafin2 make a unique pairing with respect to their homologues. However, considering that the interaction between these proteins forms the central focus of the manuscript,

© 2021. Published by The Company of Biologists under the terms of the Creative Commons Attribution License (https://creativecommons.org/licenses/by/4.0/).

it is suggested that the authors take this opportunity to more closely define the nature of this interaction based on the sequence similarities/difference amongst the homologues.
Figure 5 presents new data on JIP4 mutants. In line with the data referenced in Figure 3, the Phafin2-Binding-Region is critical to target JIP4 to tubules and is not conserved in JIP3. The chimera experiment reported in Figure 5E and 5F highlight that JIP3 is only capable of tubule targeting if given the Phafin-binding region of JIP4, while JIP4 targeting is abrogated if given the equivalent region in JIP3. As we elaborate in the discussion, the vast majority of currently described binding partners of JIP4 and JIP3 use other highly conserved regions and are therefore unsuitable to confer selectivity.

5. Line 217, Fig. 7G-I: The authors' findings regarding increased intracellular Dextran levels do not correspond with data presented in ref #17 in what I understand to be largely the same experiment. Is this a cell-type specific effect, or is there an alternate explanation?
In Figure 4, we report data in both RPE1 cells and HT1080 cells (which were used in ref #17), and have ablated JIP4 using both a knockout and with siRNA. It does not appear to be a cell-type specific effect.

6. No working model is presented to summarise the key findings of the study.
The Discussion section highlights each cluster of findings in this study, as well as how they might fit into existing literature. We have also summarised key findings to conclude the Discussion.

Minor points:

D is missing in Figure 2

Some form of quantification should be provided in figure 3E and F Supplementary Fig. 1 has display panels A-D, which are not matched by the legend.

These points have been addressed in the revised manuscript.

Reviewer #3

In the manuscript entitled "JIP4 is recruited by the phosphoinositide-binding protein Phafin2 to promote recycling tubules on macropinosome", Kia Wee Tan and colleagues show that the adaptor protein JIP4 physically interacts with the PI3P-binding protein Phafin2. The authors indicated the sites of interaction between Phafin2 and JIP4 as sites on macropinosomes where recycling tubules originate. This interaction is required for JIP4 localization on macropinosomes and is specific for JIP4, since its homologue JIP3 does not bind to Phafin2 and is not recruited to macropinosomes. In addition, they show that PI3P is required for the localization of Phafin2 and hence of JIP4 on macropinosomes. The authors also generated a JIP4-KO cell line that they used with live and fixed samples to perform functional studies on JIP4 that led the authors to the conclusion that JIP4 is involved in recycling from macropinosomes, since in the absence of JIP4 the cargo they used to track macropinosomes (10KD dextran) is retained within intracellular organelles.

Major comments:
The authors build their manuscript on JIP4 on their previous observation that Phafin2 is a novel regulator of macropinosome formation (ref 16.) However, this observation is only available at bioRxiv as an unreviewed manuscript since 2017 (Schink, K.O., et al., The PtdIns3P-binding protein Phafin2 escorts macropinosomes through the cortical actin cytoskeleton. 2017, bioRxiv) Is there a more updated or different and reviewed version of these data?
The Phafin2 manuscript has been revised following reviewer comments, but neither the previous nor the currently submitted Phafin2 manuscript have any alterations in the data that pertain to this JIP4 manuscript. We will provide the revised manuscript if requested.
While the work reported here on JIP4 originated in previous work on Phafin2 and the text makes occasional reference to the behaviour of Phafin2, these localizations are shown in this JIP4 manuscript independently. The dual localization of Phafin2 is seen in Figure 1G, 1H, Movie 1, and discussed as a minor point in Discussion. The PtdIns3P-dependence of Phafin2 is shown in Figure 1I and 1J, expected from its FYVE domain, and reported in the literature independently from our work (Matsuda-Lennikov et al., 2014; T.-X. Tang et al., 2017; T. X. Tang et al., 2020). The Phafin2 manuscript on bioRxiv contains some data from a two-hybrid screen with Phafin2 as bait. These will

© 2021. Published by The Company of Biologists under the terms of the Creative Commons Attribution License (https://creativecommons.org/licenses/by/4.0/).

be removed from the revised version of the bioRxiv Phafin2 manuscript and are instead included in the present JCS manuscript as Supplemental Data Table S1.

While the interaction of JIP4 and Phafin2 is nicely studied and reported, the major problem I see concerns the conclusion that JIP4 localizes on tubules and promotes tubulation and recycling from macropinosomes. In fact, tubules are not clearly visible in any of the images reported in the Figures. These are visible only for some of the JIP4-positive structures in suppl. Video1. Instead, the figure images clearly show that the colocalization of JIP4 with macropinosomes is restricted to specific domains. In many instances it is difficult to discriminate whether these domains pertain to the macropinosome surface or are instead endosomes tethered to/fusing with macropinosomes. The authors should provide much more convincing evidence of JIP4 association with/effect on tubulation at macropinosomes.
In the revised manuscript, we have more clearly documented the localization of JIP4 to macropinosome subdomains. Movie 3 has been added which shows JIP4 as it accumulates on macropinosomes into regions from which membrane tubules emerge. Figure 5 shows several images and quantifications that JIP4 is at macropinosome tubules. Figure 6 and Figure S6 show correlative electron microscopy data that JIP4 accumulations are visible as tubules extruded and continuous with the parent macropinosome. These extended tubules are much too long to be fusing endosomes, and the timelapse montages presented in Figure 6 and S6, as well as in Figure 7F document that they originate from the macropinosome.

The functional studies on JIP4-KO cells do not result in any conclusive evidence since a block of macropinosomal recycling should lead to an increase in macropinosome area, which is not reported among the phenotypes induced by JIP4-KO. The authors instead describe that the cargo they use to trace macropinocytosis, i.e. 10KD dextran, accumulates in JIP4-KO cells 30 min after internalization. Here there are three points the authors should/could consider:

1. The accumulation of dextran occurs in compartments that do not appear, judging by the images, to be macropinosomes but rather endosomal structures. Higher resolution images corroborated by morphometric analysis are necessary
See reply to Comment #2.

2. 70KD dextran (internalized exclusively by macropinocytosis) would be preferable to 10KD dextran since the latter can be internalized also by fluid phase and clathrin-mediated endocytosis. The use of EIPA to discriminate macropinocytosis from endocytosis is questionable since EIPA inhibits other forms of internalization under certain conditions (Canton J, Front Immunol 9: 2286). In addition the authors do not show the impact of JIP4 depletion on macropinocytosis (as the number of macropinosomes/cell), and they do not clearly demonstrate that the higher amount of intracellular dextran is due to defective recycling rather than increased internalization. Furthermore, given the expertise of the authors with live imaging, the visualization of JIP4-positive tubules carrying the cargo (dextran) would add strong evidence that the JIP4-positive tubules are actually used for the recycling of cargos. For example, dextran recycling and release by WT and JIP4-KO cells could also be measured by TIRF microscopy. In addition, the functional role of JIP4 in cargo recycling from early macropinosomes has been tested in WT and JIP4-KO cell lines. In this case it would be beneficial for the claim and for the specificity of JIP4-mediated cargo recycling to perform a rescue experiment in JIP4-KO cells by overexpressing JIP4.
Figure 4 now also reports data using 70kDa dextran in RPE1 and HT1080 cells. In addition to JIP4 knockout, we have also included data using siRNA knockdown of JIP4, and in both cases, JIP4 ablation is associated with increased retention of dextran. VAMP3 is shown exiting the macropinosome in JIP4-positive tubules in Figure 7F, S7, and Movie 4. We also report in Figure 4C and 4D that rates of macropinocytosis and size of macropinosomes formed are not changed in JIP4 knockout cells. In the Discussion, we have also qualified that our data is consistent with a macropinosome recycling defect, but offered also a possible alternative interpretation of increased intracellular dextran. In addition, the revised manuscript is clearer that dextran serves as a marker for internalized fluid, not that the fluid itself is a JIP4 cargo.

3. They should at the same time follow an endocytic tracer to exclude an impairment in endocytic (and not specific macropinocytic) recycling.
We do not make the claim that JIP4 is specific only to macropinosomes. Our data are mainly derived from macropinocytic carriers and we therefore restrict our claim to macropinocytosis.

© 2021. Published by The Company of Biologists under the terms of the Creative Commons Attribution License (https://creativecommons.org/licenses/by/4.0/).

Moreover, since both clathrin-dependent vesicles and macropinosomes mature into endosomes or endosome-like compartments, it is probable that these vesicles behave similarly.

Although the authors show by different means the physical and functional interaction of Phafin2 and JIP4, most of the data lack quantitative analysis and statistical evaluation of the results (see Fig.3E, F; 4A, B; Fig.5 and Fig.6 and see minor comments).
In most cases they show colocalization of JIP4 with other markers by plotting the fluorescence intensities of the two proteins in a Region of Interest without providing any quantitative or statistical analysis, the results shown in Fig.3E, F; 4A, B; Fig.5 and Fig.6 cannot be more than observations.
The figures in the revised manuscript now include quantitative analysis alongside example images.

A general consideration regards the use of a single cell line (RPE1) and a single JIP4-KO line (RPE-JIP4-KO) that raises concerns about how general (or cell-specific) are the claims proposed in the manuscript. Testing the model described in the manuscript in a different cell line and, at least, in two other JIP4-KO clones (or in RPE1 treated with siRNAs against JIP4) would add substantial strength to the manuscript. The suggested supplemental experiments are fully in line with the expertise of the authors and with the methods already applied in the manuscript and can be completed in 3-6 months.
The revised manuscript includes data obtained in RPE1 and HT1080 cell lines, as well as siRNA knockdown of JIP4.

Minor comments:

Fig. 1B-C-D: error bars and statistical analysis should be added.

Fig. 1E: Number of replicates and statistical analysis has to be added.

Fig. 1F: the authors should include the WB with anti-GFP or anti-JIP4 to show immunoprecipitation efficiency of the bait.
The above has been addressed in the revised manuscript.

Fig. 2C: The use of Rapamycin at 250nM for 30 minutes can reduce mTOR activity and thus induce autophagy. To exclude any interference of the mTOR/autophagic pathway, the authors should test Rapamycin at lower concentration/shorter time of treatment or include in the manuscript (as supplemental data) the evaluation of mTOR activity by measuring phosphorylation of S6-Kinase in RPE either untreated and treated with 250nM Rapamycin.
SAR-405 inhibition of VPS34 would inhibit induction of autophagy. It is unavoidable that Rapamycin will inhibit mTOR at this concentration, but it does not impact the conclusions of this relocalization assay. JIP4 has not been reported to localize to mitochondria.

Fig. 3C-D: error bars and statistical analysis should be added.

Fig. 3E-F: Number of replicates, quantitative and statistical analysis has to be added.

Fig. 3F: scale bar is missing.

Fig. 4A, E: Number of replicates, quantitative and statistical analysis has to be added.

Fig. 5: Quantitative analysis is missing.

Fig. 6: Number of replicates, quantitative and statistical analysis has to be added.
The above points have all been addressed in the revised manuscript.

Fig. 7G: EIPA is not mentioned either in the main text or in methods or in figure legend.
The use of EIPA has now been explained in main text and methods.

Fig. 7E, H: as suggested in the major comments, JIP4-overexpression in WT cells and in JIP-4KO cell has to be performed to confirm JIP4 activity on dextran recycling.
siRNA experiments have been added in Figure 4.

© 2021. Published by The Company of Biologists under the terms of the Creative Commons Attribution License (https://creativecommons.org/licenses/by/4.0/).

REFERENCES FOR REVIEWERS

Bonet-Ponce, L., Beilina, A., Williamson, C. D., Lindberg, E., Kluss, J. H., Saez-Atienzar, S., . . . Cookson, M. R. (2020). LRRK2 mediates tubulation and vesicle sorting from lysosomes. *Sci Adv, 6*(46). doi:10.1126/sciadv.abb2454

Kerr, M. C., & Teasdale, R. D. (2009). Defining Macropinocytosis. *Traffic, 10*(4), 364-371. doi:https://doi.org/10.1111/j.1600-0854.2009.00878.x

Marchesin, V., Castro-Castro, A., Lodillinsky, C., Castagnino, A., Cyrta, J., Bonsang-Kitzis, H., . . . Chavrier, P. (2015). ARF6–JIP3/4 regulate endosomal tubules for MT1-MMP exocytosis in cancer invasion. *211*(2), 339-358. doi:10.1083/jcb.201506002

Matsuda-Lennikov, M., Suizu, F., Hirata, N., Hashimoto, M., Kimura, K., Nagamine, T., . . . Noguchi, M. (2014). Lysosomal interaction of Akt with Phafin2: a critical step in the induction of autophagy. *PLoS One, 9*(1), e79795. doi:10.1371/journal.pone.0079795

Schink, K. O., Tan, K. W., Spangenberg, H., Martorana, D., Sneeggen, M., Campsteijn, C., . . . Stenmark, H. (2017). The PtdIns3P-binding protein Phafin2 escorts macropinosomes through the cortical actin cytoskeleton. *bioRxiv*, 180760. doi:10.1101/180760

Tang, T.-X., Jo, A., Deng, J., Ellena, J. F., Lazar, I. M., Davis, R. M., & Capelluto, D. G. S. (2017). Structural, thermodynamic, and phosphatidylinositol 3-phosphate binding properties of Phafin2. *Protein Science, 26*(4), 814-823. doi:https://doi.org/10.1002/pro.3128

Tang, T. X., Finkielstein, C. V., & Capelluto, D. G. S. (2020). The C-terminal acidic motif of Phafin2 inhibits PH domain binding to phosphatidylinositol 3-phosphate. *Biochim Biophys Acta Biomembr, 1862*(6), 183230. doi:10.1016/j.bbamem.2020.183230

Williamson, C. D., & Donaldson, J. G. (2019). Arf6, JIP3, and dynein shape and mediate macropinocytosis. *Molecular Biology of the Cell, 30*(12), 1477-1489. doi:10.1091/mbc.E19-01-0022

****

<u>Second decision letter</u>

MS ID#: JOCES/2021/258495

MS TITLE: JIP4 is recruited by the phosphoinositide-binding protein Phafin2 to promote recycling tubules on macropinosomes

AUTHORS: Kia Wee Tan, Viola Nahse, Coen Campsteijn, Andreas Brech, Kay Oliver Schink, and Harald Stenmark
ARTICLE TYPE: Research Article

Thank you for sending your manuscript to Journal of Cell Science through Review Commons.

Thank you for your careful revisions, I am happy to tell you that your manuscript has been accepted for publication in Journal of Cell Science, pending standard ethics checks.

<u>Reviewer 1</u>

Advance summary and potential significance to field

This study identifies a Phafin2-JIP4 interaction at the heart of the connection between the cytoskeleton and cargo recycling in the dynamic process of pinocytosis. A better understanding of these proteins might lead to a broader understanding of the dynamics of these short-lived vesicles and how they regulate other critical signal transduction pathways.

© 2021. Published by The Company of Biologists under the terms of the Creative Commons Attribution License (https://creativecommons.org/licenses/by/4.0/).

Comments for the author

The authors have satisfactorily addressed previous concerns.

Reviewer 2

Advance summary and potential significance to field

The paper identifies two novel components (JIP4 and Phafin2) of the molecular machinery mediatimg the tubular recycling pathway that operates from macropinosomes.

Comments for the author

The authors have satisfactorily addressed my comments.

© 2021. Published by The Company of Biologists under the terms of the Creative Commons Attribution License (https://creativecommons.org/licenses/by/4.0/).
